# Cardiovascular Response to Posture Changes: Multiscale Modeling and *in vivo* Validation During Head-Up Tilt

**DOI:** 10.3389/fphys.2022.826989

**Published:** 2022-02-17

**Authors:** Matteo Fois, Simona Vittoria Maule, Marta Giudici, Matteo Valente, Luca Ridolfi, Stefania Scarsoglio

**Affiliations:** ^1^Department of Mechanical and Aerospace Engineering, Politecnico di Torino, Turin, Italy; ^2^Autonomic Unit, Department of Medical Sciences, Università Degli Studi di Torino, Turin, Italy; ^3^Department of Environmental, Land and Infrastructure Engineering, Politecnico di Torino, Turin, Italy

**Keywords:** gravity, orthostatic stress, head-up tilt table testing, computational hemodynamics, multiscale cardiovascular modeling

## Abstract

In spite of cardiovascular system (CVS) response to posture changes have been widely studied, a number of mechanisms and their interplay in regulating central blood pressure and organs perfusion upon orthostatic stress are not yet clear. We propose a novel multiscale 1D-0D mathematical model of the human CVS to investigate the effects of passive (i.e., through head-up tilt without muscular intervention) posture changes. The model includes the main short-term regulation mechanisms and is carefully validated against literature data and *in vivo* measures here carried out. The model is used to study the transient and steady-state response of the CVS to tilting, the effects of the tilting rate, and the differences between tilt-up and tilt-down. Passive upright tilt led to an increase of mean arterial pressure and heart rate, and a decrease of stroke volume and cardiac output, in agreement with literature data and present *in vivo* experiments. Pressure and flow rate waveform analysis along the arterial tree together with mechano-energetic and oxygen consumption parameters highlighted that the whole system approaches a less stressed condition at passive upright posture than supine, with a slight unbalance of the energy supply-demand ratio. The transient dynamics is not symmetric in tilt-up and tilt-down testing, and is non-linearly affected by the tilting rate, with stronger under- and overshoots of the hemodynamic parameters as the duration of tilt is reduced. By enriching the CVS response to posture changes, the present modeling approach shows promise in a number of applications, ranging from autonomic system disorders to spaceflight deconditioning.

## 1. Introduction

Despite the influence of gravity on the human cardiovascular system (CVS) has been recognized and studied for decades (Coonan and Hope, 1983; Blomqvist and Stone, 2011; Smith et al., 1994; Hall and Hall, 2020), several aspects still need to be understood. Among the most intriguing issues, the key role played by autonomic and autonomous regulatory mechanisms in maintaining global system homeostasis under the action of gravity—or their inefficient intervention due to pathological conditions—deserves further investigation. These aspects are crucial to comprehend how regulation and adequate blood pressure and perfusion levels are preserved during active and passive orthostatic stress (i.e., when standing still or lying on a rotating table tilted to an almost vertical position, respectively). Meanwhile, a number of autonomic system disorders or dysautonomias, such as orthostatic hypotension, orthostatic intolerance, and syncope (Goldstein, 2017), can affect the CVS response to gravity stress. Such problems are also of great importance to space medicine, engaged in devising new strategies to counteract autonomic deconditioning following long-term exposure to the space environment (Clément, 2011; Gunga et al., 2016). Within this framework, head-up tilt (HUT) and tilt-down table tests serve as fundamental diagnostic and prognostic clinical tools and are widely employed to study and monitor dysautonomia occurrence in patients (Cheshire and Goldstein, 2019).

Cardiovascular mathematical modeling has been proven to provide reliable and feasible alternatives to *in vivo* measurements in describing overall CVS functioning (Ottesen et al., 2004; Westerhof et al., 2010; Quarteroni et al., 2017). The adaptability of CVS models can help understand the basic principles of the cardiovascular response under different working and pathological conditions such as hemorrhages, cardiac arrhythmias, valve diseases, etc. (Liang et al., 2009a,b; Reymond et al., 2009; Blanco and Feijóo, 2013; Broomé et al., 2013; Scarsoglio et al., 2014; Mynard and Smolich, 2015). Single cause-effect mechanisms can be identified, isolated, and reproduced, choosing suitable levels of personalization and spatial detail (ranging from 0D to 3D reconstructed geometries), enabling the design of innovative bio-engineering solutions to treat cardiovascular diseases. The explicit effect of gravity onto multiscale CVS modeling has been accounted for only in recent years. In their seminal works, Heldt et al. (2002) and Heldt (2004) designed a multi-compartment lumped parameter model of the human circulation, simulating orthostatic stress induced by tilt table test and including baroreflex and cardio-pulmonary reflex control onto blood pressure. Then, a number of researchers resorting to similar strategies followed: Artiles et al. (2016) analyzed the effect of artificial gravity (centrifugation) and exercise (cycling) onto global hemodynamics; Olufsen et al. (2005) and Williams et al. (2014) focused on autonomic and autonomous (cerebral) regulation of blood pressure and flow, and onto patient-specific modeling of HUT, respectively; van Heusden et al. (2006) discussed the importance of vascular stress-relaxation in delaying lower limbs venous filling. Recent high-dimensional models were aimed at reproducing cardiovascular alterations promoted by posture changes exploiting 1D and 3D representations of the whole CVS (Zhang et al., 2017) (though excluding short-term regulation), or of specific regions of interest, e.g., the aortic arch (Lau and Figueroa, 2015). To the best of our knowledge, no multiscale models of the complete circulation, including arterial, microvascular and venous networks, pulmonary and cardiac compartments, and proper regulatory mechanisms have been proposed to study passive orthostatic stress, so far.

In this picture, our work has a 2-fold aim: (i) to present a novel 1D-0D closed loop model able to describe complex wave pressure and flow patterns along the arterial tree, systemic microcirculation and venous return before and after HUT, and (ii) to use this model to investigate systematically the transient response to HUT under different tilting rates.

Our multiscale and multi-compartment layout includes a 1D description of the large systemic arteries and 0D analogs of the systemic venous, arteriolar, venular, and capillary beds compartments, as well as of the superior and inferior venae cavae and cardio-pulmonary circulation. In this way, wave propagation phenomena are represented along the 1D arterial tree, whereas the 0D circuital models of the remaining peripheral microcirculation and venous return are organized into five distinct body regions, from head to feet, to account for the role of gravity onto different body pools. Autonomic regulation mediated by baroreflex response, cardio-pulmonary reflex, and autonomous regulatory systems, such as cerebral autoregulation, have been included only separately in past works, but never as a whole, to the best of our awareness. Differently, submodels of baroreflex control (Ottesen et al., 2004), cardio-pulmonary reflex (Heldt, 2004), and cerebral autoregulation (Ursino and Lodi, 1997) were implemented together into our model, providing continuous pursuing of system homeostasis. Venous valves were introduced along arms and legs venous compartments to prevent reverse flow (Mynard et al., 2012). Intrathoracic—intrapleural, extra-chamber (Heldt, 2004)—and intracranial pressures—cerebrospinal fluid (Ursino and Lodi, 1997; Holmlund et al., 2018)—, within pertaining extravascular environments, were included to account for their influence on the system during posture changes.

A thorough validation of the model was conducted, by making a detailed comparison between model outcomes and an extensive literature collection of more than forty clinical works. *In vivo* transient and pressure waveform validation was also performed, considering a cohort of fourteen young healthy volunteers. The clinical protocol included steady-state brachial and continuous finger (with reconstruction of brachial waveform) arterial blood pressure and heart rate measurement, with an estimation of associated hemodynamics parameters, while undertaking HUT tests to 30° and 70°. The proposed model was used to investigate in detail short-term hemodynamic alterations following passive tilt to upright posture. The steady-state and transient model response was assessed in terms of main hemodynamic parameters, including mean arterial pressure (*MAP*), heart rate (*HR*), stroke volume (*SV*), cardiac output (*CO*), total peripheral resistance (*TPR*), central venous pressure (*CVP*), cardiac work and oxygen consumption indexes, regional blood volumes, pressure, and flow rate waveform analysis. A systematic study of the CVS response to increasing tilting rate from supine to 70° HUT, and *vice versa* for the tilt-down case, was proposed.

## 2. Materials and Methods

### 2.1. Mathematical Model

**Figure 1:**
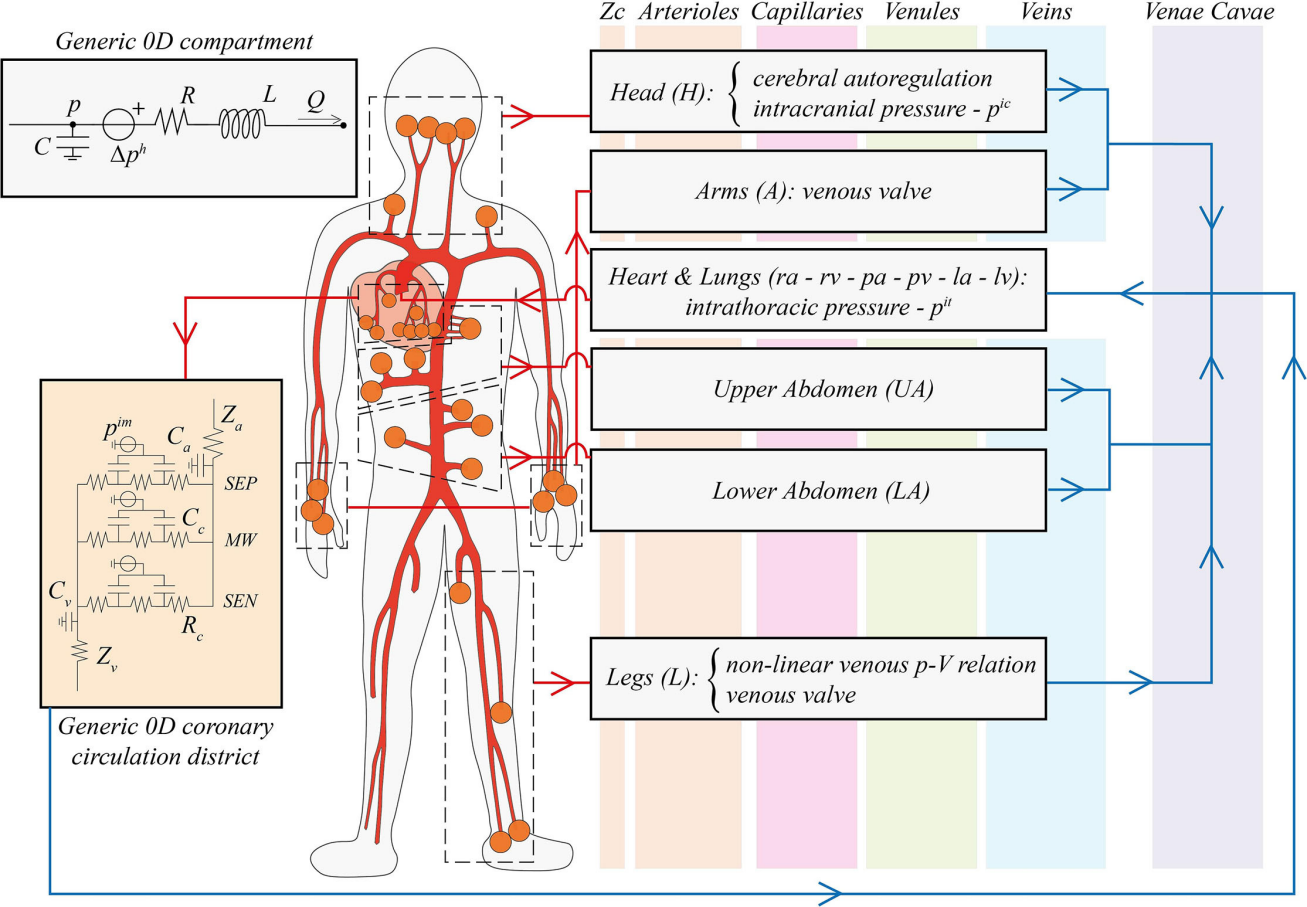
Schematic illustration of the global multiscale cardiovascular model. The thick red network represents the 1D arterial tree, while the vertical colored bands (orange, pink, green, and blue, from left to right) refer to the different compartments of the 0D systemic circulation (from arterioles to venae cavae, respectively). Orange circles indicate a 1D arterial connection with distal 0D arterioles, organized in black dashed boxes by different body regions (head, arms, upper and lower abdomen, legs, plus the coronary circulation). In the right grey boxes, the subdivision of the 0D systemic circulation is reported, specifying improvements of our work associated with gravity alterations: vascular control of cerebral autoregulation, the intracranial pressure *p*^*ic*^, the intrathoracic pressure *p*^*it*^, arms and legs venous valves, and the non-linear pressure-volume relationship for leg veins. The *Heart* & *Lungs* 0D compartment includes the right atrium (*ra*) and ventricle (*rv*), pulmonary arteries (*pa*) and veins (*pv*), and the left atrium (*la*) and ventricle (*lv*). Red arrows denote arterial links, whereas blue arrows indicate venous connections. In the top left panel, a generic 0D Windkessel model is depicted: *p* and *Q* denote blood pressure and flow rate, *R*, *C*, and *L* are compartmental resistance, compliance, and inertance, Δ*p*^*h*^ is the hydrostatic pressure contribution due to gravity. In the orange left panel, a generic 0D coronary microcirculatory district is reported: *SEP*, *MW*, and *SEN* identify myocardial layers, *Z*_*a*_, *Z*_*v*_, *C*_*a*_, *C*_*v*_ are arterial and venous input/output impedances and compliances, respectively, *R*_*c*_ and *C*_*c*_ are intra-layer resistances and compliances, and *p*^*im*^ is the intramyocardial pressure.

The present model of the human CVS is an extension of the previous works by Guala et al. (2015), Gallo et al. (2020), and Gallo et al. (2021). The global layout is composed of a 1D description of the arterial tree and 0D electric analogs of the remaining systemic microvasculature (arterioles, capillaries, and venules), the venous return (veins and venae cavae), and the cardio-pulmonary circulation. In the following, the model is described from a functional and conceptual point of view, by focusing on the primary physiological features taken into account (a scheme of the model is reported in Figure 1), while mathematical and numerical details are reported in the Supplementary Material, where Supplementary Figure 1 illustrates the multiscale configuration.

**1D Arterial Tree**. The main arteries were modeled as straight vessels with circular cross-section shape, originating from the aortic valve and splitting into smaller branches through successive bifurcations, as shown in Figure 1. Vessels tapering was taken into account, and the network geometry is reported in Supplementary Table 1. Blood motion was described by the 1D axisymmetric form of the Navier-Stokes equations for mass and momentum balance (Supplementary Equations 1, 2), with dependent variables *A*(*x, t*) and *Q*(*x, t*) representing vessel lumen and blood flow rate, respectively, (*t* and *x* are the time and vessel axial coordinate, respectively). Through vessel lumen *A*(*x, t*), transverse deformation was accounted for, according to vessel wall mechanical properties, whereas no longitudinal stretching or contraction was admitted. Blood was modeled as a Newtonian fluid and a flat-parabolic velocity profile is assumed over each cross-section area. Mechanical and viscoelastic (vessel-dependent) properties of the arterial walls were reproduced by including an additional constitutive equation for pressure *p*(*x, t*) (Supplementary Equation 3) to close the 1D mathematical system. Gravity action was included considering vessels orientation γ with respect to the frontal transverse body axis, and the tilt angle α, corresponding to vessels inclination from the horizontal reference.

As boundary conditions for the 1D problem, mass and total pressure conservation were imposed at inlet/outlet sections of vessels associated with arterial bifurcations (Supplementary Equations 4, 5). External conditions applied at the entrance of the aorta by coupling the proximal aorta with a 0D model of the aortic valve, and at each terminal branch by plugging a 0D arteriolar compartment (through a set of lumped characteristic impedances) to each terminal 1D artery. The method of characteristics was adopted to deal with each boundary value problem (Guala et al., 2015).

**0D Systemic microvasculature and venous return**. Systemic distal circulation, microcirculation, and venous return were represented as lumped parameter compartments. A distinct arteriolar circuital model was included for each 1D terminal artery. Arteriolar compartments were then grouped into five major regions: head, arms, upper abdomen, lower abdomen, and legs, as illustrated in Figure 1. For each region, one capillary, one venular, and one venous compartment was defined. Three additional districts were included to represent superior, inferior, and abdominal venae cavae, directly connected to the cardio-pulmonary circulation. The subdivision of the 0D systemic circulation into five distinct regions represents the major layout improvement with respect to the last versions of Gallo et al. (2020, 2021). In this way, the gravitational effects onto different body portions can be properly studied, devoting special focus to the head and legs compartments, where the largest gravity impact occurs.

Lumped parameter compartments were organized as 3-elements RLC Windkessel models (an example of a generic 0D compartment is reported in the top panel of Figure 1), apart from RLCR arteriolar compartments, including the associated characteristic impedances (RLC layouts were preferred to simple RC ones to account also for blood inertia). The corresponding governing equations (Supplementary Equations 6–8, 10) involve compartmental intraluminal pressure *p*(*t*), blood flow rate *Q*(*t*), and compartmental total blood volume *V*(*t*). This latter depends on the compartmental transmural pressure and the associated compliance *C*, according to a linear pressure-volume constitutive law. Transmural pressure was obtained as the difference between intra- and extra-vascular pressure, where the latter corresponds to either intrathoracic, intracranial, intramyocardial, or environmental pressure, depending on the specific compartment. For the legs venous compartment, a different constitutive law (Supplementary Equation 9) was introduced to resemble non-linear effects of distending veins volume, when subject to high pressure levels caused by gravitational stress (Melchior et al., 1994; Heldt et al., 2002; Heldt, 2004; Lim et al., 2013). Parameter setting was based on previous modeling works (Heldt, 2004; Liang et al., 2009b; Lim et al., 2013; Gallo et al., 2020, 2021). Particular attention was devoted to total blood volume repartition over the global system. A total blood volume of 5,700 ml was assumed (Gallo et al., 2020), then blood was distributed according to physiological proportions reported in Karpeles and Huff (1955); Leggett and Williams (1991); Hall and Hall (2020), and Wayson et al. (2018).

Gravity contribution was accounted for by including a gravity source term (represented as a lumped pressure generator), depending on hydrostatic pressure gradients, within the 0D compartments, as proposed in Heldt et al. (2002); Heldt (2004); Lim et al. (2013), and Artiles et al. (2016). However, previous models did not consider arteriolar and venular compartments besides arterial and venous ones. In our model, instead, arteriolar, capillary and venular compartments are present and describe very confined vascular districts, which are not affected by gravitational stress when undergoing posture variations. Therefore, hydrostatic pressure gradients were included only within venous and venae cavae compartments, due to their intrinsic anatomical extension with respect to the longitudinal body axis. Hydrostatic pressure contribution was expressed according to Stevino's law (Supplementary Equation 11), involving blood specific weight, the hydrostatic height of the corresponding fluid columns, and the relative orientation with respect to the horizontal reference (the tilt angle α). Hydrostatic heights of blood columns were associated with the anatomical length of the corresponding compartments projected along the longitudinal body axis.

**0D Cardio-pulmonary circulation**. The cardio-pulmonary circulation (Supplementary Equations 12–16) was modeled as described in our previous works (Gallo et al., 2020, 2021). For each cardiac chamber, transmural pressure was linked to the corresponding stressed volume by means of a time-varying elastance, *E*(*t*). This latter function depends upon the corresponding maximum elastance amplitude and baseline value, respectively, through the normalized elastance function *e*(*t*), with a different formulation for atria and ventricles. Cardiac valves (Supplementary Equations 17, 18) were represented as non-ideal diodes accounting for several effects onto valve leaflets: tissues friction, pressure and inertial forces, and the influence of downstream vortexes. Each 0D valve model included the so-called Bernoulli's resistance of the valve—accounting for convective acceleration and dynamic pressure losses—as well as valve viscous resistance and inertance. The model dependent variable is the blood flow rate, and the opening state depends on the pressure gradient across the valve.

Pulmonary circulation was represented through an arterial and a venous compartment, each including a lumped resistance and a compliance, according to a 2-element Windkessel configuration.

**Intrathoracic pressure**. Intrathoracic pressure improves venous return mechanisms by promoting cardiac preload, i.e., right atrium filling (Buda et al., 1981; De Cort et al., 1993; Klabunde, 2016; Pstras et al., 2016; Yartsev, 2016; Verhoeff and Mitchell, 2017). As intrinsically influenced by gravity-induced alterations, intrathoracic pressure was included in the current model as a novelty with respect to Gallo et al. (2020, 2021). As suggested by Heldt (2004) and Artiles et al. (2016), at a fixed posture intrathoracic pressure was here superimposed as an external (extra-vascular or extra-chamber) and constant pressure acting onto thoracic compartments (cardiac chambers and pulmonary circulation). Intrathoracic pressure is believed to decrease by a few mmHg when assuming upright posture, due to gravity gradient onto thoracic cavity fluids (Mead and Gaensler, 1959; Bryan et al., 1966; Yartsev, 2021). Following Heldt et al. (2002); Heldt (2004), intrathoracic pressure variation during HUT was defined as a cosinusoidal function of time (Supplementary Equations 19, 20).

**Multiscale coronary circulation**. A multiscale description of the coronary circulation was included in our model, distinguishing between a 1D representation of the largest coronary vessels and a 0D circuital model of the downstream coronary microcirculatory districts, perfusing the different layers of the cardiac muscle. To this aim, the model introduced by Mynard et al. (2014); Mynard and Smolich (2015) was adopted (as shown in circuital scheme in the orange left panel of Figure 1). Each 0D microcirculatory district was subdivided into three branches referred to each myocardial layer (subepicardium, midwall, and subendocardium), further composed of an arterial, an intermediate, and a venous compartment. The detailed mathematical description of the multiscale coronary model is reported in Saglietto et al. (2022).

**Venous valves**. Venous valves ensuring unidirectional flow were modeled as non-ideal diodes as introduced by Mynard et al. (2012), and also adopted by Keijsers et al. (2015, 2016) and Zhang et al. (2017). The venous valve model is an additional improvement of the current work with respect to our previous versions and was integrated within legs and arms venous compartments. Valves intervention was described through non-linear resistances and inertances associated with those compartments (Supplementary Equations 21–24). Each venous valve was virtually placed in between the end of the corresponding venous compartment and the following vena cava.

**Baroreflex model**. Short-term autonomic control mechanisms were included in the model following Gallo et al. (2020, 2021). The baroreceptor model (Supplementary Equations 25–27) accounts for the inotropic effect of both ventricles, the chronotropic effect due to the heart rate regulation, as well as the control of the systemic vasculature (peripheral arterial resistances, unstressed volume of the venous system, and venous compliance). Tuning and calibration of the parameters controlling sympathetic and parasympathetic activity were made in order to match reported behaviors encountered during posture changes (Coonan and Hope, 1983; Blomqvist and Stone, 2011; Smith et al., 1994). Sympathetic and parasympathetic activities were calculated as a function of the mean aortic-carotid sinus pressure (average aortic arch, right and left carotid sinus pressure) with respect to its target value. For arterial baroreflex, the assumed target pressure corresponds to the supine mean aortic-carotid sinus pressure.

Cardio-pulmonary reflex (Heldt, 2004; Lim et al., 2013) was introduced in the same fashion as previously described for arterial baroreflex (Supplementary Equations 28, 29). Low-pressure receptors are located into the right atrium, in order to perceive the central venous pressure (*CVP*). Cardio-pulmonary target pressure was therefore assumed equal to supine right atrium mean pressure, and sympathetic/parasympathetic activities were computed as a function of the current mean right atrial pressure over the target value (that is, the supine mean right atrial pressure). Arterial baroreflex efferent organs control equation applied also for cardio-pulmonary reflex, but only peripheral resistances and venous tone were affected.

**Cerebrovascular system and cerebral autoregulation**. Cerebrospinal fluid pressure (i.e., intracranial pressure, Supplementary Equations 30–33) was modeled based on Davson's equation (Holmlund et al., 2018), regarding cerebrospinal fluid resistance and rate of formation, and dural veins pressure, which was taken equal to *CVP* (right atrium pressure) in supine position. Assuming Davson's equation holds for any body position, a similar expression can be derived also for the tilted posture, where dural veins pressure was approximated as the *CVP* at tilted posture minus the head-right atrium hydrostatic contribution obtained according to Stevino's law. Here, body position relative to the horizontal reference also applied. Even for modest tilt angles, the cerebrospinal fluid system is no longer a unique fluid media, due to venous collapse caused by the zero transmural pressures at jugular level. This aspect was also implemented in the model, by including the hydrostatic gradient associated with the fluid column extending from the head to the jugular point of collapse at zero transmural pressure (here the jugular vein was considered as corresponding to the superior vena cava compartment). Collapse tilt angle α_*collapse*_ is the inclination for which the jugular venous pressure—i.e., superior vena cava pressure—approaches zero. Due to the low compliances of arteriolar, venular and capillary compartments, intracranial pressure was introduced only within cerebral veins, as illustrated in Figure 1. This led to the maintenance of cerebral veins transmural pressure at negligible levels even at elevated tilt angles, agreeing with findings regarding the non-collapsibility of such vessels (Leggett and Williams, 1991; Hall and Hall, 2020; Kosugi et al., 2020), together with their non-deformable status enabled by the cranial box structure.

Cerebral autoregulation—ensuring constant cerebral blood flow (*CBF*) through brain vessel vasoconstriction/dilation—was implemented following the model by Ursino and Lodi (1997) (Supplementary Equations 34–39) as further control mechanism involved in gravity alteration dynamics, with respect to the last versions by Gallo et al. (2020, 2021). The key-point of the model is to define cerebral arteriolar compliances and resistances as non-linear function of the mismatch between the current overall *CBF* and a given reference value (corresponding to the supine mean overall *CBF*). Cerebral arteriolar compliances varied between bounding maximum and minimum values, whereas arteriolar resistances were determined exploiting Poiseuille's law.

**Numerical simulation**. 1D governing equations were discretized and solved numerically according to a Discontinuous Galerkin Finite Elements approach and integrated in time employing a 2-step Runge-Kutta explicit scheme (Gallo et al., 2020) with constant time step. Time integration of 0D governing equations was achieved *via* the same 2-step Runge-Kutta explicit scheme (the 1D-0D coupling scheme is provided in the Supplementary Material). Numerical simulations were carried out in MATLAB 2020b environment.

### 2.2. Simulating Head-Up Tilt Test

Tilt test simulations were based on Heldt (2004) modeling approach (Supplementary Equation 40): tilt angle α introduced in the model equations was computed as a cosinusoidal function of time, starting from the supine position and reaching the final tilted posture within a given rotation period. Three different tilt angles were selected for the investigation, 30°, 70°, and 90°. All levels of inclination were reached keeping a constant tilting rate. Then, in order to investigate its role in CVS response, different tilting rates were explored in the range from 1.4 to 35°/s.

**Figure 2:**
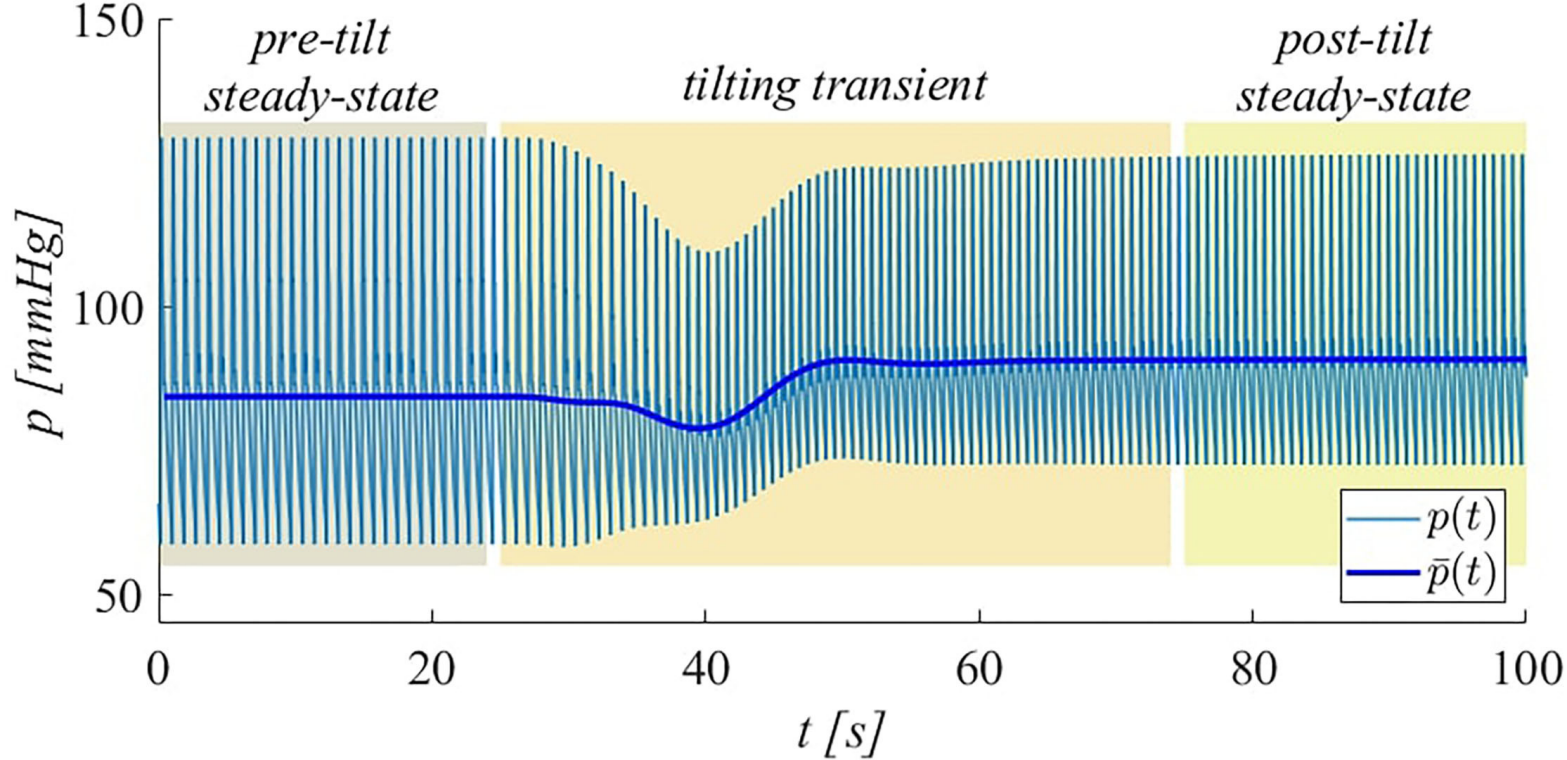
Illustration of simulated brachial pressure behavior during HUT to 70° at 4°/s. Both instantaneous (*p*) and beat-averaged ($\bar{p}$) pressure signals are shown. The intervals corresponding to pre-tilt steady-state, tilting transient, and post-tilt steady-state are highlighted.

In our model, pre- and post-tilt steady-state conditions were defined such that initial and post-tilt transient responses can be assumed as completely extinguished. The tilting transient is the interval between the two (pre- and post-tilt) steady-state conditions. As an example, the typical (brachial) pressure signal obtained during HUT to 70° is displayed in Figure 2, where the three intervals (pre-tilt steady-state, tilting transient, and post-tilt steady-state) are identified.

### 2.3. *In vivo* Head-Up Tilt Test

To provide *in vivo* validation of the proposed model, clinical measurements were carried out at the *Città della Salute e della Scienza* Hospital in Turin, at the “Autonomic Unit” laboratory of the Department of Medical Sciences, University of Turin. Monitored hemodynamic variables included heart rate and non-invasive arterial pressure measurements performed in several sites.

**Candidates selection and ethical approval**. Fourteen healthy male volunteers were enrolled and tested, all fulfilling age (20–30 yo), weight (50–95 kg), and height (160–200 cm) requirements. We focused on male subjects to be coherent with the model setup, which is calibrated for a generic healthy male individual. Anthropometric features of subjects are listed in Table 1. The clinical study conformed to the standards set by the Declaration of Helsinki, except for registration in a database. The study was evaluated and approved by the local ethic committee (Comitato Etico Interaziendale A.O.U. Città della Salute e della Scienza di Torino-A.O. Ordine Mauriziano-A.S.L. TO1-CEI/330). All subjects provided their written informed consent to participate in this study.

**Table 1:** Anthropometric features of subjects (BMI is the body mass index, computed as Weight/Height^2^, whereas μ and σ denote the mean and SD values).

**Subject**	**Age [yr]**	**Weight [kg]**	**Height [cm]**	**BMI [kg/m**^**2**^**]**
1	26	60	175	19.6
2	26	73	175	23.8
3	26	78	182	23.5
4	25	78	190	21.6
5	26	82	175	26.8
6	26	74	171	25.3
7	26	92	198	23.5
8	29	70	187	20.0
9	23	75	186	21.7
10	26	80	175	26.1
11	28	68	178	21.5
12	27	75	182	22.6
13	29	80	178	25.2
14	24	82	180	25.3
μ	26.2	76.2	180.9	23.3
σ	1.7	7.5	7.3	2.2

**Medical equipment and facilities**. Continuous heart rate and finger arterial pressure recordings were obtained *via* the Finapres Finometer MIDI device (FMS, 2021), with Height Correction Unit enabled and reconstruction of brachial arterial pressure signal (Guelen et al., 2003). Through the same instrument, also stroke volume, cardiac output and total peripheral resistance were estimated. Two digital sphygmomanometers were employed to capture punctual heart rate and systolic, mean and diastolic arterial pressure at brachial and calf level. To perform tilt table tests, a standard TTPD2-AV-HD dynamic bed was used, with a nominal tilting velocity of about 10° for every 2.5s.

**Experimental protocol**. Finometer finger/brachial continuous pressure recordings and punctual sphygmomanometer measurements were conducted over all subjects enrolled in the experimental campaign. The experimental protocol consisted of two separate steps of tilting: first with the subject reaching an inclination of 30° starting from the supine position, afterward, with the subject returned again to the supine position, tilting to 70°. Each level of tilt was approached after 5 min of Finometer basal recording with the subject lying in supine position. Tilted posture was subsequently maintained for 5 min of Finometer continuous recording. In this way, the complete transient response to the change of posture was tracked, and the new steady-state condition was captured, with respect to the previous basal status. Punctual pressure and heart rate measurements were performed at each steady-state condition (supine, 30° and 70° HUT) by means of digital sphygmomanometers as a term of comparison for the Finometer continuous measurements (heart rate, brachial, and calf pressures were averaged through three measurements). During tilt, all subjects were instructed neither to speak nor to contract their legs muscles, in order to resemble actual passive standing and not favor venous return. Since respiration may largely affect long-term oscillations extent within pressure signals, subjects were asked to avoid deep breathing during recording.

## 3. Results

### 3.1. Model Validation

The present model was validated following a 2-fold approach, considering both the steady-state and transient response to passive change of posture. Firstly, steady-state pre- and post-tilt model outcomes (e.g.„ *MAP, HR, SV, CO*, blood volume shift, brachial pressure waveforms) were compared to *in vivo* data both reported in the literature and measured in our HUT experiments. Second, model transient response to passive tilt was compared with our *in vivo* recordings.

**Steady-state response validation**. More than 40 studies were considered (full literature references can be found in the Supplementary Tables 7a,b) and clinical works were preferred to modeling ones. Due to the importance of aortic blood pressure, only its invasive catheterized measures (or reconstructions *via* transfer function) were considered as reference.

Results are reported in Table 2, showing the supine baseline condition against the steady-state hemodynamic response to two modeled levels of tilt, i.e., 30° and 70°. Literature references are reported as absolute or relative ranges (with respect to supine baseline condition). Comparisons with our *in vivo* HUT measures, when present, are reported in square brackets. Since literature data do not report significant differences between actual standing at 90° and 70° HUT, only the latter case is reported in Table 2 (in the Supplementary Material, both cases are included as a term of comparison). As shown in Table 2, the model well reproduces the global hemodynamic response to passive change from supine to upright posture. In particular, relative variations with respect to the supine configuration fall within literature ranges. Almost all parameters also match available literature absolute ranges. The only exception regards our *in vivo SV, CO* and *TPR* data, which were overestimated by the Finometer due to its limited absolute accuracy, despite being capable of capturing parameters variation (Chin and Panerai, 2012) due to posture changes.

**Table 2:**
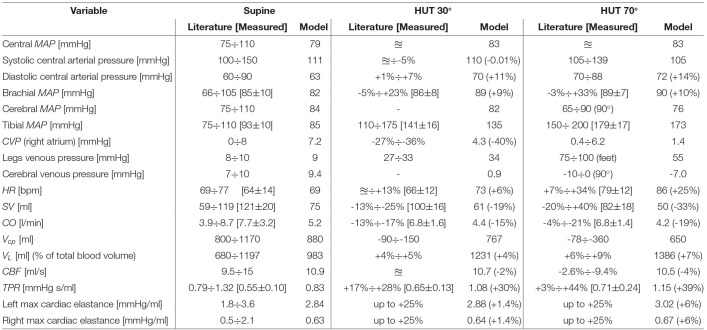
Comparison between literature data (≊: no significant variation, − : no data available), our *in vivo* findings (in square brackets), and model outcomes for steady-state supine, HUT 30° and 70°.

*MAP mean arterial pressure, CVP central venous pressure, HR heart rate, SV stroke volume, CO cardiac output, V_cp_ cardio-pulmonary blood volume, V_L_ legs blood volume, CBF cerebral blood flow, TPR total peripheral resistance. Literature references are reported in the Supplementary Material*.

The heterogeneous multi-compartment nature of our model allows for the representation of gravity alterations onto both arterial and venous side of the CVS, resulting in physiological increase in pressure distribution along the head-feet direction, in line with most literature references.

One remarkable consequence is the difference between central and brachial (mean) arterial pressure, which is often considered as interchangeable with the subject lying supine, but that is no more the same when undergoing posture changes, due to the difference in height between the heart and the cuff measurement on the arm. This height mismatch results in a central mean arterial pressure little augmented (+5%) over all tilted positions, despite a higher increase in mean brachial pressure with the tilt angle (+9% at 30° HUT, +10% at 70°), also confirmed by our *in vivo* measurements. One possible interpretation for the modest central mean arterial pressure increase observed at a tilted posture (in both the literature and model outcomes)—with respect to the supine basal level—can be found in the mean aortic-carotid sinus pressure targeted by the baroreflex control system. In fact, such mean pressure is mostly affected by the reduced blood pressure sensed at the carotid sinus level, triggering an augmented active control onto efferent organs.

Central systolic and diastolic pressures also mirror behaviors reported in the literature and recorded by our *in vivo* measurements, with the former slightly reduced with increasing tilt angle (different behaviors can be found in the literature, with systolic pressure trend varying widely with posture changes), whereas the latter appears as markedly raised in all cases. Simulated *CVP* (i.e., right atrium pressure) varies in accordance with literature findings, falling to a value as low as 1.4 mmHg when approaching upright posture (70° HUT), driven by the drop in intrathoracic pressure. As expected from literature evidence, modeled cerebral venous pressure falls below zero (relative to external environmental pressure), although no collapsibility of cerebral vessels occurs thanks to the cerebrospinal fluid action discussed above (cerebral venous transmural pressure is maintained close to zero). In addition, modeled *CBF* is almost perfectly conserved over all simulated positions, as a result of cerebral autoregulation intervention. Another feature of our model is its capability of reproducing typical blood shift from the upper (cardio-pulmonary) to lower (buttocks, legs) regions of the body. As reported in Table 2, about 400 ml of blood is transferred from the upper (cardio-pulmonary circulation, abdomen) to lower pools, mostly within venous compartments, due to their higher compliance.

**Waveform validation**. Additional steady-state validation is provided through modeled brachial pressure waveform comparison with experimental signals extracted by the Finometer. A number of 50÷60 (depending on samples length) consecutive brachial pressure waveforms were acquired, normalized in time (with respect to each heartbeat RR duration), and averaged for each volunteer. Then, the ensemble average pressure waveform was obtained by averaging all 14 subject-specific waves, rescaled with respect to their time-averaged mean pressure value. SD bands were computed including both intra- and inter-subject variances, as detailed in the (Supplementary Equation 41). The corresponding simulated signals were extracted from the brachial artery for the model supine basal and HUT 70° steady-state configurations.

**Figure 3:**
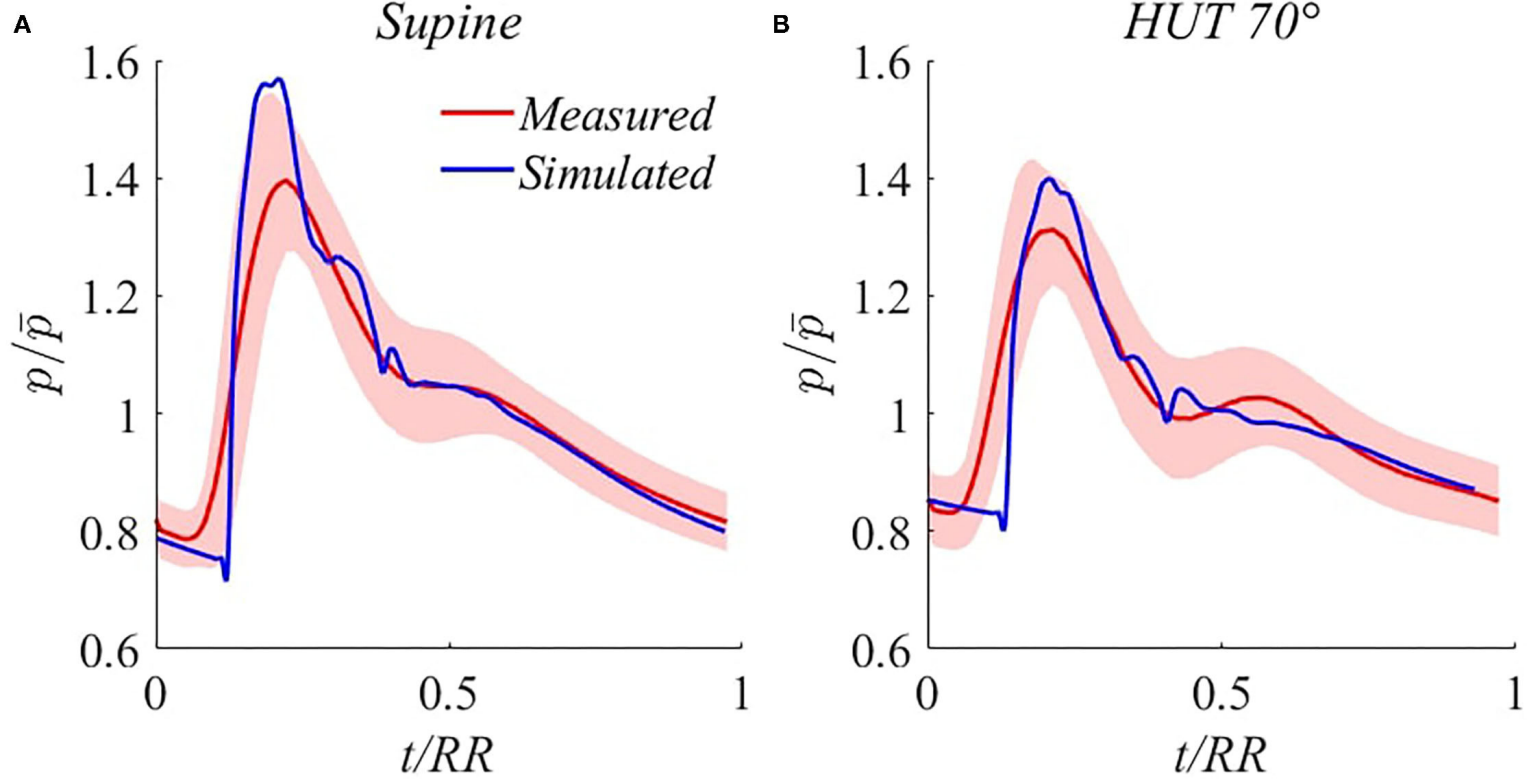
Comparison between simulated (blue) and measured (red, with SD bands) steady-state brachial pressure waveforms for supine **(A)** and 70° HUT **(B)**. $\bar{p}$ denotes brachial *MAP*.

As reported in Figure 3, traveling waves phenomena (transmission, reflection, and superposition of forward and backward waves) along arterial tree are accurately reproduced by our model. In particular, brachial pressure waveform reshaping following HUT involves mainly the amplitude of the signal, i.e., the pulse pressure, which is markedly reduced, in agreement with the reduction in systolic pressure accompanied by a net increase in diastolic pressure. The dicrotich notch and the secondary peak position appear as delayed approximately to the same extent for the simulated and measured waveforms, and the simulated wave irregularities are always comprised within the SD bands of the measured average signal, except for the foot of the wave. This slight discrepancy is likely induced by the smoothing due to the averaging of the measured signals, with consequent overestimation of diastolic and underestimation of systolic pressure.

**Figure 4:**
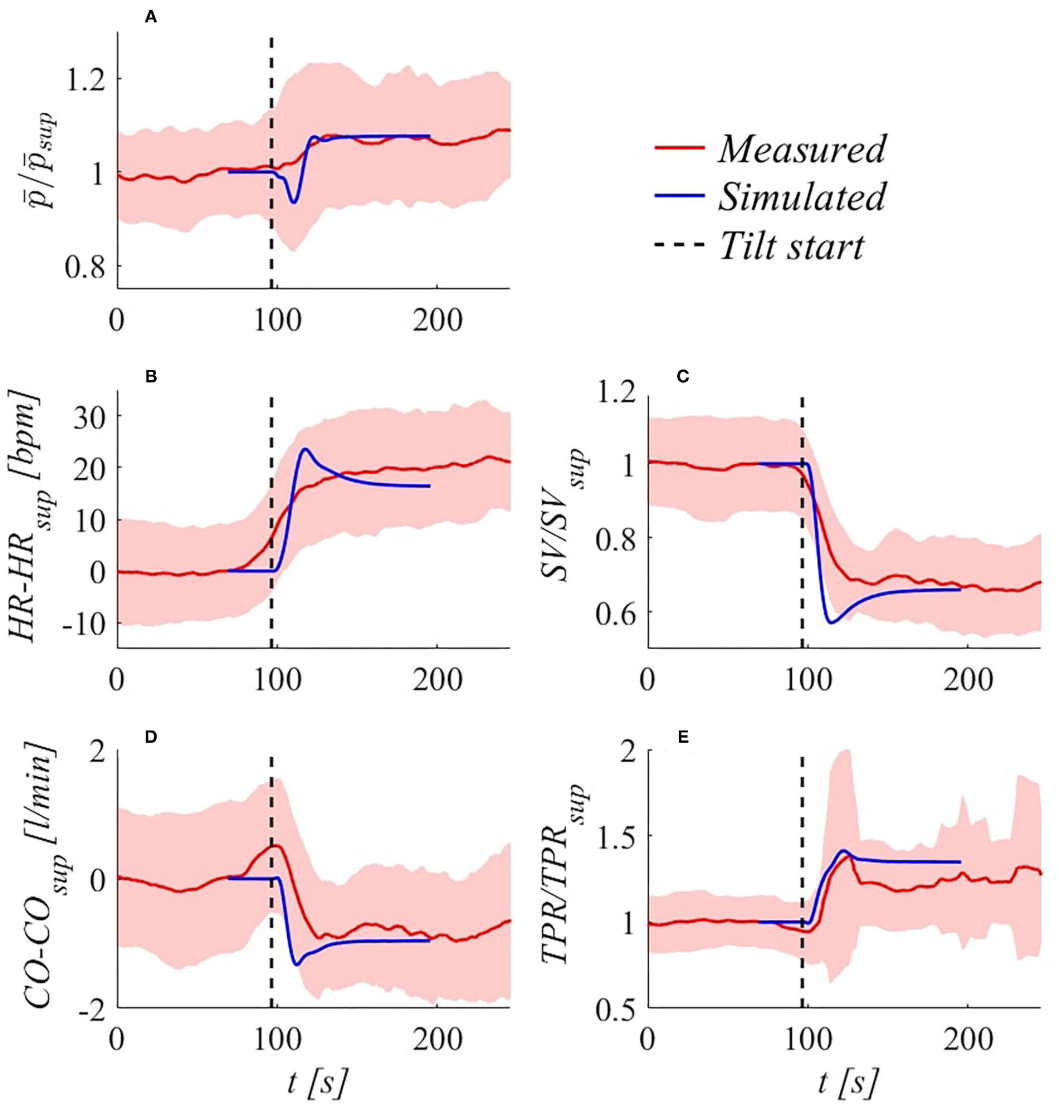
Simulated (blue) and experimental (red, with SD bands) transient hemodynamic response following 70° HUT at 4°/s: **(A)** brachial $\bar{p}$/${\bar{p}}_{sup}$ ($\bar{p}$ denotes brachial MAP), **(B)** *HR*−*HR*_*sup*_, **(C)** *SV*/*SV*_*sup*_, **(D)** *CO*−*CO*_*sup*_, **(E)** *TPR*/*TPR*_*sup*_. Subscript *sup* refers to supine condition.

**Transient response validation**. The model is able to accurately reproduce transient responses following change of posture for the most common hemodynamic parameters—e.g.„ *MAP, HR, CO, SV*, and *TPR* refer to Critchley et al. (1997); Toska and Walløe (2002); Youde et al. (2003); Heldt (2004); Olufsen et al. (2005); van Heusden et al. (2006); Truijen et al. (2012); Sundblad et al. (2014); Williams et al. (2014); Lau and Figueroa (2015), and Ishbulatov et al. (2020)—both reported in the literature and recorded in our *in vivo* measurements. Figure 4 shows beat-to-beat hemodynamic parameters adjustment to HUT, reaching upright posture (70°) within 17.5 s (4°/s). Results are referred to the supine position, in terms of ratio or absolute difference depending on the considered variable.

Very good agreement is found between experimental and model outcomes (taking into account inter-subject variability), in particular for brachial *MAP* (denoted as $\bar{p}$), whose initial drop caused by sudden fluid migration from upper to lower regions (and consequent reduction in ventricular filling) is well recovered by means of regulation mechanisms intervention. Simulated under- and overshoots are largely in line with observations reported in other studies (Toska and Walløe, 2002; Heldt, 2004; Williams et al., 2014). Notice that subjects averaging tend to smooth strong fluctuations that are not in phase among subjects, as can be observed considering *HR* sequence (Figure 4B). Nevertheless, SD band peaks are well comparable with modeled over- and undershoots for $\bar{p}$, *SV, CO*, and *TPR* sequences (Figures 4A,C–E).

### 3.2. Steady-State Results

The proposed model was used to study in detail the effects of passive HUT onto the CVS. Results presented in this section are organized by describing first a global picture of the most relevant steady-state behaviors following tilt to 70°, throughout the 1D arterial tree up to 0D venous return compartments. Then, the role of tilting rate onto the transient response to 70° HUT is systematically investigated.

**Figure 5:**
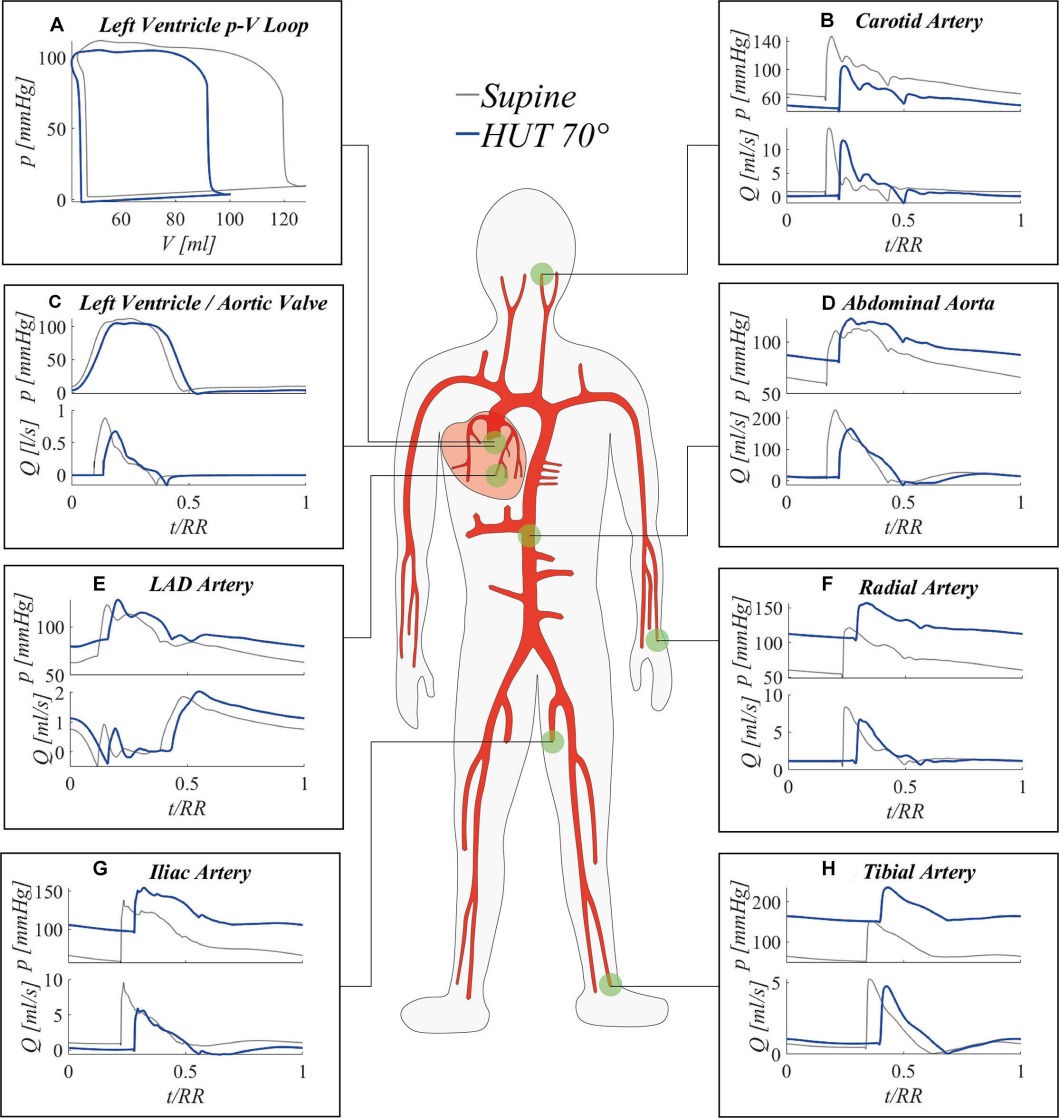
Simulated steady-state results at 70° HUT (blue) and in the supine position (gray): **(A)** left ventricle pV loop; pressure (top) and flow rate (bottom) signals at left ventricle **(C)**, and at representative sites along the arterial tree **(B,D–H)**.

Figure 5 shows steady-state pressure and flow rate time signals (normalized with respect to their RR heartbeat duration) in seven body sites, including the left ventricle and six locations along the arterial tree. As the main consequence of the increase in *HR* following tilt to 70°, post-tilt signals are shifted out of phase—with respect to the supine condition—ue to the modification of the systole-diastole ratio within the single beat duration. In fact, as the *HR* rises, the systolic phase tends to be favored with respect to diastole, resulting in a different balance of the two cardiac phases onto the overall shape of the wave. In general, flow wave amplitude reduction reflects a drop in *CO* typically observed in the literature (Coonan and Hope, 1983; Blomqvist and Stone, 2011; Smith et al., 1994) during the short-term response to the change of posture, with flow mean value diminishing significantly up to −20% at the aortic valve (Figure 5C) and all along the aorta (bottom diagrams of Figures 5D–H). Pressure signals are clearly shifted up or down depending on the positive or negative effect of gravity (top diagrams of Figures 5B,D–H), due to the hydrostatic contribution associated with the column of blood measured from the heart to each site. This contribution entails arterial mean pressure values more than doubled at the tibial level, as confirmed by previous clinical studies (Coonan and Hope, 1983; Blomqvist and Stone, 2011; Smith et al., 1994) and our *in vivo* measurements (shown in Table 2). Conversely, mean pressure reduction is experienced at carotid/cerebral level (Figure 5B), causing a change in mean cerebral perfusion pressure (i.e., mean cerebral pressure minus intracranial pressure) and eliciting activation of autoregulation mechanisms. Due to heart chambers emptying following downward blood shift, left ventricle end-diastolic volume is considerably decreased, leading to a marked reduction of the stroke work (*SW*, area of the left ventricle pressure-volume loop, Figure 5A), which is a proxy of the cardiac energy supply.

**Figure 6:**
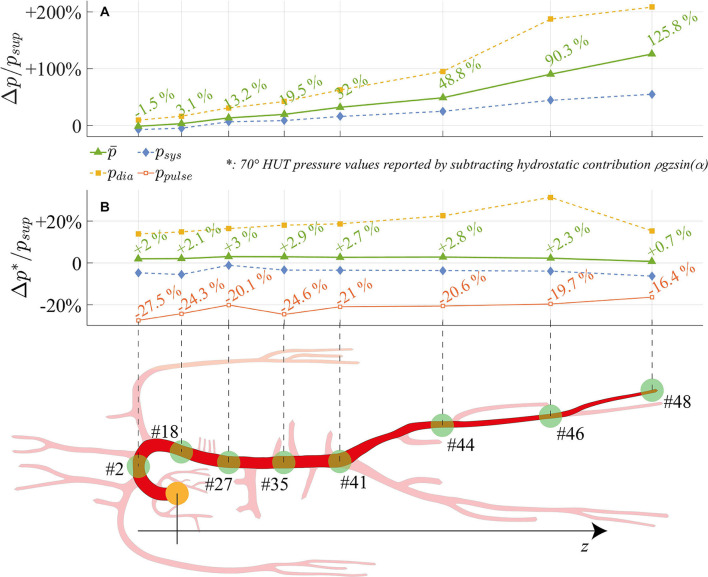
Relative variation between 70° HUT and supine condition for steady-state pressures (systolic, mean, diastolic, pulse pressure) along the aorta and legs large arteries: the hydrostatic contribution is **(A)** taken into account (Δ*p*/*p*) and **(B)** neglected (Δp^*^/*p*). *z* is the longitudinal body axis.

Figure 6 offers a closer perspective of pressure waveform alterations following tilt to 70°. By focusing on eight arterial sites (from the aortic arch down to the lower extremity of the posterior tibial artery) mean, systolic and diastolic pressures relative differences between 70° HUT and supine position are presented. Pressure changes are depicted both taking into account (top panel Figure 6A, 70° HUT-to-supine pressure variations Δ*p*) and subtracting (bottom panel Figure 6B, Δp^*^) the hydrostatic contribution.

The top panel Figure 6A shows how systolic, mean, and diastolic pressures rise as one moves away from the heart, eventually reaching values more than doubled for the mean pressure (+125.8% at feet level). Only at the summit of the aortic arch a reduction in mean pressure is registered, diminished by 1.5% with respect to the supine condition. In presence of the hydrostatic contribution (Figure 6A), diastolic pressure increases more than systolic in the feetwards direction. When the hydrostatic contribution is removed (Figure 6B), diastolic pressure is still markedly augmented with respect to its supine value, by a value as high as +20% on average. Despite the global reduction in venular and venous compliance accompanied by the augmented total peripheral resistance, we recall that diastolic pressure rise is enhanced (beside the effect of gravity) by the different interplay between systole and diastole following increased cardiac rhythm (*HR*), which tends to lengthen the former phase leaving then ventricles shorter time to relax. Conversely, systolic pressure is little reduced along the whole arterial system. Mean pressure undergoes only small alteration (+0.7%÷+3%), following a non-monotonic trend from head to feet, with a peak at the thoracic level. Results displayed in Figure 6 reveal the importance of the hydrostatic contribution onto mean pressure variation connected to HUT differently from the central region, the hydrostatic contribution alone represents almost the entire alteration at the lower extremities. The reason for such behavior may be found in the regulatory action of the baroreflex mechanisms, together with the effect of intrathoracic pressure reduction onto venous return and cardiac filling, both of which influence mostly central arterial vessels.

In Figure 6B, relative variations of pulse pressure with respect to supine values are also reported. As expected from clinical evidences (Coonan and Hope, 1983; Blomqvist and Stone, 2011; Smith et al., 1994), pulse pressure is reduced along the entire aorta at 70° HUT, especially due to the enhanced diastolic pressure. Pulse pressure partially recovers along the feetwards direction, probably because of the combined effect of the stiffening and vasoconstricting regulatory action driven by the baroreflex control, and the wave reflection phenomena typically occurring at peripheral sites. The overall pulse pressure drop mirrors the reduction in cardiac work (*SW*, see panel a of Figure 5) by 33% at 70° HUT, which highlights the different working conditions faced by the CVS when undergoing passive HUT.

**Figure 7:**
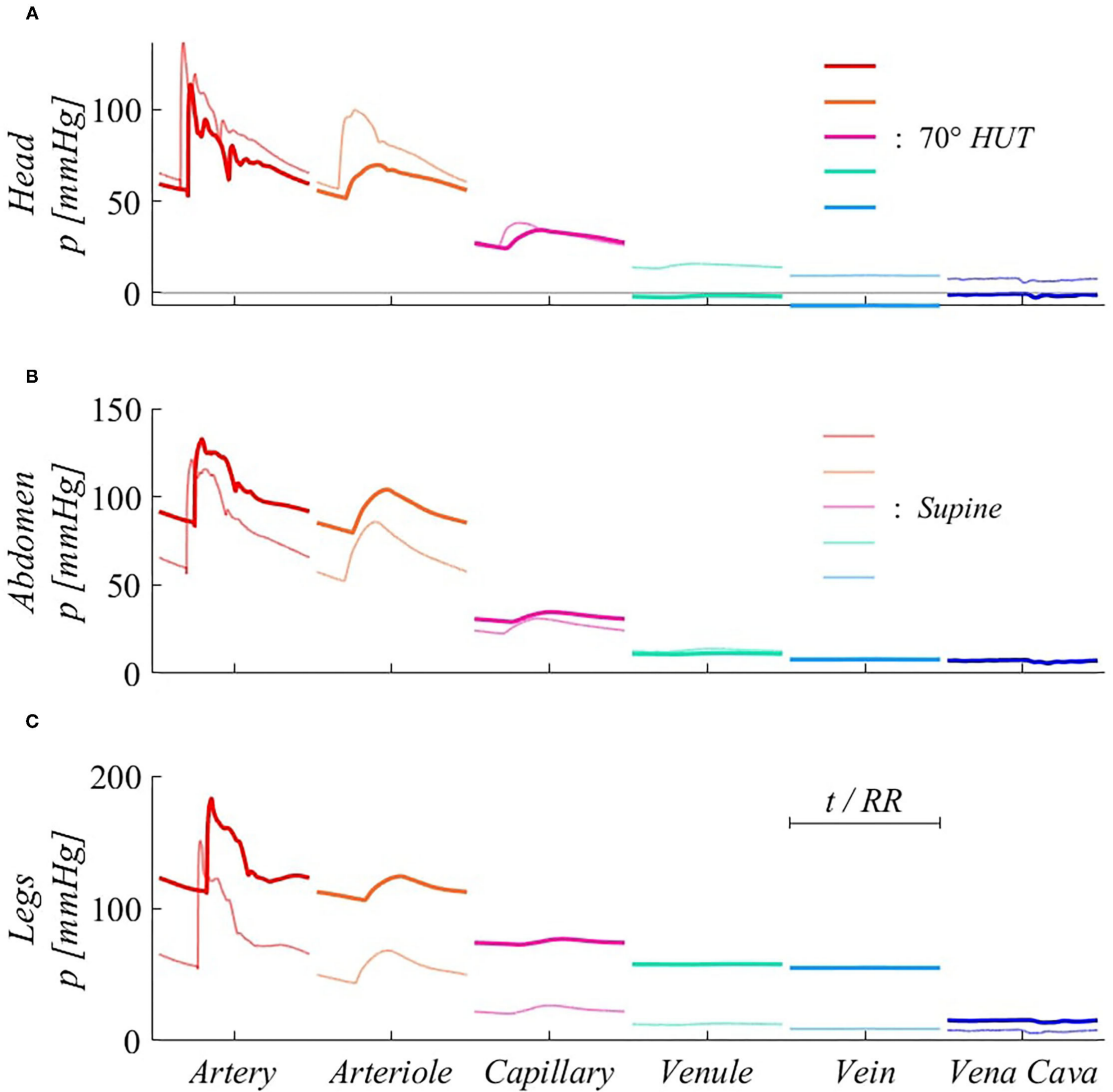
Steady-state pressure signals at supine (thin, light colored lines) and 70° HUT (thick, dark colored lines) for the 0D side of the systemic circulation, from terminal arteries to venae cavae 0D compartments. Panels refer to different body regions: **(A)** head (artery #13), compartmental midpoint at *z* = −0.15 m; **(B)** abdomen (artery #34), compartmental midpoint at *z* = +0.15 m, **(C)** legs (artery #45), compartmental midpoint at *z* = +0.72 m (distances are measured from the right atrium position along the longitudinal body axis *z*).

Figure 7 focuses mostly on the 0D components of the model, describing systemic distal arterioles and capillary beds, and systemic venous return throughout venular, venous, and venae cavae compartments. Three representative body regions are chosen to describe gravity effects onto such side of the circulation: head, abdomen, and legs (Figures 7A–C). For 0D compartments, the reported pressure signals refer to the inlet point of each corresponding circuital analog. Steady-state pressure signals are presented for each 0D compartment, after time normalization with respect to the heartbeat duration *RR*. The different interplay between systole and diastole following *HR* variation also here induces signals phase shifting, as already evidenced in Figure 5.

Pressure variations along the systemic pathway of Figure 7 are ruled by resistive effects, which are important in microvascular compartments (arteriole, capillary), and hydrostatic gradient effects, which are instead negligible in the systemic microcirculation (arteriole, capillary, and venule), as these compartments are not characterized by relevant anatomical length. The strongest pressure drops occur at the arteriolar and capillary levels, both in supine and tilted conditions. In fact, arteriolar and capillary compartments provide the highest vascular resistance, since they are responsible for lowering blood pressure to a value suitable for gas exchange with organs and the pulmonary environment. For this reason, pressure drops occurring between these compartments are present both under and without the effect of gravity.

A further relevant drop emerges between veins and venae cavae compartments at 70° HUT for the legs region (Figure 7C). Such pressure drop is instead exclusively due to hydrostatic gradient effects. In fact, between leg veins and the following abdominal vena cava compartment, a vertical distance of approximately 0.5 m is encountered, causing the reported 40 mmHg (hydrostatic) pressure drop. Almost no such pressure drop is registered at abdominal level (Figure 7B), since abdominal venous compartments have no dominant vertical dimension. Head veins (Figure 7A) experience negative intraluminal pressure after reaching 70° HUT, because of their position above the heart, although their tone does not vary significantly thanks to the peculiar extravascular condition (Blomqvist and Stone, 2011; Leggett and Williams, 1991; Holmlund et al., 2018; Hall and Hall, 2020; Kosugi et al., 2020).

### 3.3. Transient Response Results

**Figure 8:**
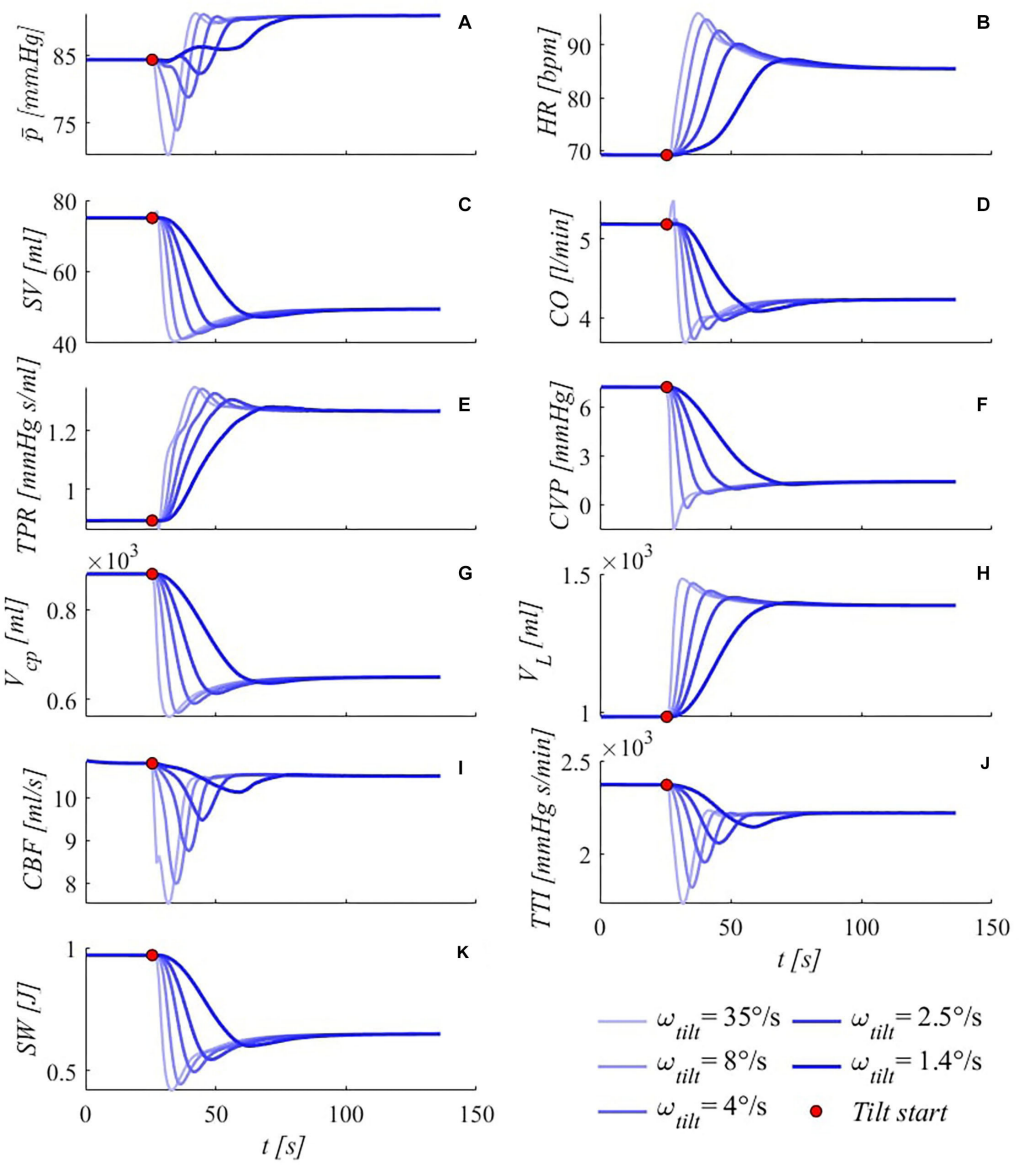
Simulated transient response of some meaningful hemodynamic parameters to passive HUT from supine position to 70°, for five different tilting rates (35, 8, 4, 2.5, and 1.4°/s from light thin to dark thick blue lines). **(A)** brachial mean arterial pressure $\bar{p}$, **(B)** heart rate *HR*, **(C)** stroke volume *SV*, **(D)** cardiac output *CO*, **(E)** total peripheral resistance *TPR*, **(F)** central venous pressure *CVP* (corresponding to right atrial pressure), **(G)** cardio-pulmonary blood volume *V*_*cp*_ (blood volume of the four cardiac chambers, arterial and venous pulmonary circulation), **(H)** legs vascular volume *V*_*L*_ (mostly venous blood volume, but also arterial, arteriolar, venular and capillary), **(I)** cerebral blood flow *CBF* (overall average blood flow delivered by carotid and vertebral arteries to the brain), **(J)** tension-time index *TTI* (product of the mean left ventricle pressure and RR beat duration per minute, TTI is an index of oxygen consumption), **(K)** stroke work *SW* (area of the left ventricle pressure-volume loop). Red points denote tilting starting point.

The transient response of the model to a specific rate of tilt (4°/s) has already been discussed in the transient response validation section (Figure 4). Here, we systematically investigate the effect of different tilting velocities to 70° HUT on the transient response of some meaningful hemodynamic parameters. In Figure 8, model outcomes under five different tilting rates—from 35°/s to 1.4°/s, chosen in the typical ranges investigated in the pertinent literature—are illustrated.

As first observation, the same steady-state solution is approached by all variables under all different rates of tilt, showing that the post-tilt steady-state configuration is independent of the tilting rate, while the transitory duration is inversely proportional to the tilting rate. Noticeable information can be gathered from both the most common (brachial *MAP*-here $\bar{p}$ -, *HR, SV, CO, TPR*, Figures 8A–E), as well as other fundamental hemodynamic parameters (Figures 8F–K). In particular, brachial mean arterial pressure $\bar{p}$ (Figure 8A) shows a non-monotonic variation for all tilting rates apart from the slowest simulated case (50 s tilting duration, with initial pressure drop almost absent), likely because of the prompt compensation mediated by the regulatory mechanisms (arterial baroreflex and cardio-pulmonary reflex). Notice that the fastest case (70° HUT reached in 2 s) may potentially be problematic for orthostatic intolerant subjects, since such a rapid rotation to upright posture—combined with the total absence of additional compensatory mechanisms (muscle pumping action of calf, legs and diaphragm)—can lead to dizziness and fainting.

In general, all variables show typical under- and overshoots, although no oscillatory behavior is observed for any of these variables, except for the mean brachial arterial pressure $\bar{p}$, where a slight peak is detected after the deep drop, in the faster tilting rates. In all cases, under- and overshoots amplitudes appear as damped in the slower tilting rates, assuming a near-monotonic approach to the steady-state configuration. An interesting behavior is observed for *CO* (Figure 8D) at the fastest tilting rate, where a peculiar initial rapid increase is detected. A possible reason for this behavior is the rapid rise in *HR* (shown in Figure 8B), overcoming the reduction in *SV* and counteracting temporarily the increase in *TPR* (shown in Figures 8C,E). This latter quantity also shows remarkable trends especially at higher tilting rates, for which the initial strong increase is suddenly decelerated by the intervention of the remaining slower efferent organs (venous tone regulation), and then fastened again to counteract abrupt effects of posture variation.

Central venous pressure (Figure 8F) decreases as a primary consequence of intrathoracic pressure falling, upon assuming an upright posture. Intrathoracic pressure drop with the tilt angle is monotone, thus the undershoot exhibited by *CVP* under fast tilt to 70° must be due to different mechanisms, such as a delay in venous return restoring. Vascular volume increase at lower limbs level (Figure 8H) and the associated decrease at central regions (Figure 8G) are widely debated, particularly concerning plausible stress-relaxation mechanisms aimed at delaying venous vascular filling (e.g., van Heusden et al., 2006). Such mechanisms may reasonably come into play at higher tilting rates, although their presence was neglected here. However, volume shifts predicted by our model result in good agreement with such clinical findings (van Heusden et al., 2006), with an amount of approximately 400 ml of fluid being transferred from upper to lower regions within 10÷60 s, even in the absence of any stress-relaxation mechanism. Cerebral autoregulatory mechanism succeeded in maintaining almost constant cerebral perfusion, with only little decrease in steady-state mean *CBF* (Figure 8I) following tilt to 70°, despite deep decrements experienced within the very early phase of tilt.

Finally, by looking at *TTI* and *SW* behaviors (Figures 8J, K), it is fairly evident how the system is engaged to balance gravity load: once the upright posture is approached, working performance (*SW*, energy supply) of the system is considerably reduced (cardiac output dropped by 19%, stroke work decreased by 33% at steady-state), in response to reduced oxygen consumption (*TTI*, energy demand), determined by the decrease in the mean left ventricle pressure (-6%) and RR interval (-20%). This implies an overall less demanding condition for the CVS functioning, due to the partial reduction in circulating blood volume (drop in *CO*) following pooling in the lower extremities. Such contractions in *TTI* and *SW* are much more evident within the first few seconds following reclination and at higher tilting rates.

## 4. Discussion

The proposed model is capable of accurately reproducing steady-state and transient responses to passive change of posture—highlighting the effect of gravity—at least for the first minutes following HUT. The main novelty of the model is the detailed multiscale and multi-compartment layout, with the 1D arterial tree connected to a 0D description of the systemic microcirculation and venous return – subdivided into five distinct body regions: head, arms, upper and lower abdomen, legs – and of the cardio-pulmonary circulation.

Results showed that gravity-induced effects trigger a number of system alterations, ranging from an increase in heart rate and peripheral systemic vascular resistance, to substantial losses of stroke volume and cardiac output (−33 and −19%, respectively, at 70° HUT), together with considerable pulse pressure contraction accompanied by cardiac work and oxygen consumption reduction. All steady-state alterations grow when the degree of tilt is augmented (as shown in Table 2 and Supplementary Tables 7a,b), and most responses are strongly non-linear with respect to the sine of the tilt angle. e.g., compare *HR* and central diastolic pressure (from Table 2): they increase non-linearly by +6 and +11% at 30° HUT and +25 and +14% at 70°, respectively. On the contrary *CVP* and blood volume shift from cardio-pulmonary compartments decrease almost linearly by –40 and –13% at 30°, and by –80 and –27% at 70°.

Blood pooling leads to the storage of a significant amount of fluid in the most compliant compartments of the lower regions, i.e., within leg veins. Additionally, baroreflex control—driven by the enhanced sympathetic activity—promotes peripheral vasoconstriction and stiffening of venular and venous compartments, by penalizing their vascular tone and raising arteriolar and capillary resistance. As a result, cardiac output drops, together with stroke volume and left ventricle pressure. The cardiac filling is therefore reduced when undergoing passive tilt to upright posture (coherently with limitation in venous return) and this likely explains the deep reduction in stroke work (–17% at 30° HUT, –33% at 70°, almost linear with the sine of the tilt angle) and oxygen consumption (*TTI* –4% at 30°, –6% at 70° HUT) experienced after the change of posture. In fact, given the strong reduction of the cardiac filling (cardiac preload)—and thus of end-diastolic left ventricular volume (stroke volume) -, cardiac pressure and stroke work are limited by the Frank-Starling mechanism of the heart (Jacob et al., 1992; Westerhof et al., 2010), which shall pump a reduced amount of blood toward the system periphery (*CO* drops by –15% at 30° HUT and –19% at 70°). Moreover, the limited *TTI* reduction is not balanced by the stronger *SW* drop, causing a temporary impairment between energy demand and supply. The whole system appears to be less stressed at (passive) upright posture than supine. We recall that passive tilt is a peculiar condition, with complete absence of muscular activation and consequent venous return enhancement. In this sense, muscular action would act as if the system was undergoing exercise conditions, even though to a limited extent, and blood return to the heart would be greatly favored. Notice that, despite substantial cardiac relaxation associated with a passive tilt to upright posture, prolonged exposure to abnormal intraluminal pressure, and excessive blood volume pooling in the lower extremities may induce severe drawbacks, such as extravascular edema, blood volume loss, pain, varicose veins insurgence, and consequent venous insufficiency, with potential cardiovascular complications (Ludbrook, 1962; Barstow and Kassop, 2019; Tansey et al., 2019), especially in the case of inefficient venous valves intervention.

From a transient point of view, the model helped understand cardiovascular acute adaptation to the passive change of posture, by means of a systematic analysis of the transient response to posture variation under different rates of tilt. The model was able to accurately predict parameters behavior, progressively exacerbated by the increasing tilting velocity (as shown in Figure 8). Results showed the occurrence of even greater fluctuations as the tilting rate increased. Near-monotonic responses to the slowest tilt were almost completely lost at faster rates, characterized by pronounced falls ($\bar{p}$, *SV, CO, CVP*, *V*_*cp*_, *CBF, TTI, SW*) and peaks (*HR, TPR*). Physiological acceptable steady-state conditions were reached for all tilting velocities, showing the combined action offered by the different regulatory mechanisms to adequately restore central pressure levels and proper system homeostasis.

**Figure 9:**
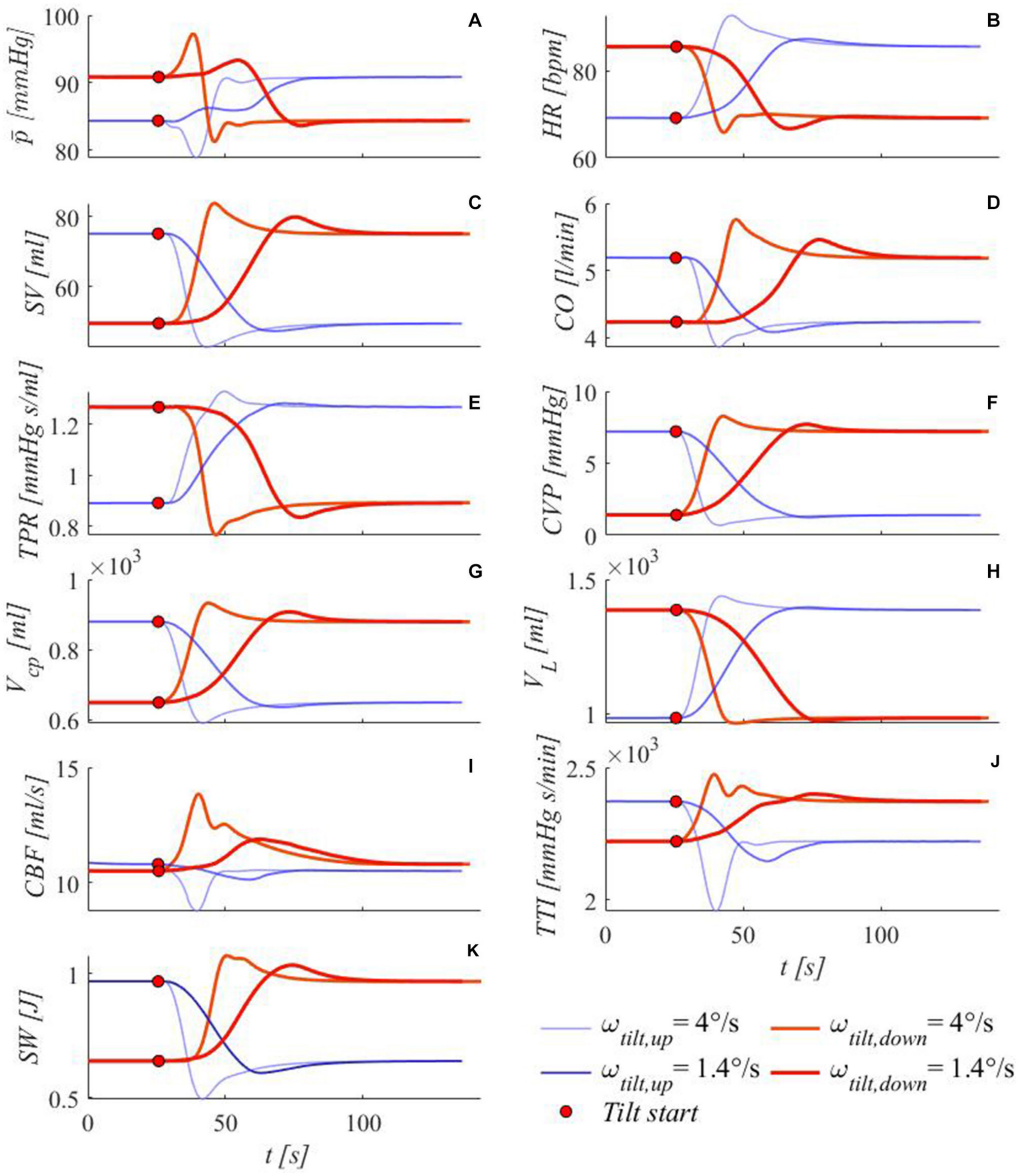
Simulated transient response of some meaningful hemodynamic variables to passive tilt-down from 70° to the supine position at two different tilting rates (red, thin lines: 4°/s, thick lines: 1.4°/s). For the sake of comparison, corresponding tilt-up transients are also reported (blue lines). **(A)** brachial mean arterial pressure $\bar{p}$, **(B)** heart rate *HR*, **(C)** stroke volume *SV*, **(D)** cardiac output *CO*, **(E)** total peripheral resistance *TPR*, **(F)** central venous pressure *CVP* (corresponding to right atrial pressure), **(G)** cardio-pulmonary blood volume *V*_*cp*_ (blood volume of arterial and venous pulmonary circulation, plus the four cardiac chambers), **(H)** legs vascular volume *V*_*L*_ (mostly venous blood volume, but also arterial, arteriolar, venular and capillary), **(I)** cerebral blood flow *CBF* (overall average blood flow delivered by carotid and vertebral arteries to the brain), **(J)** tension-time index *TTI* (product of the mean left ventricle pressure and RR beat duration per minute, TTI is an index of oxygen consumption), **(K)** stroke work *SW* (area of the left ventricle pressure-volume loop). Red points mark tilting starting point.

Several authors dealt with reverse tilt issues, from both modeling and clinical perspectives (Smith et al., 1994; Critchley et al., 1997; Toska and Walløe, 2002; Heldt, 2004; Sundblad et al., 2014). To this end, we studied the dynamic change following tilt-down from 70° to supine position, focusing on the same variables reported in Figure 8. Figure 9 reports system response following tilt-down from 70°, under two different tilting velocities (4°/s and 1.4°/s), in comparison with the corresponding tilt-up cases. In general, tilt-up under- and overshoots correspond to over- and undershoots for the tilt-down case, respectively, although with different amplitudes and times of occurrence. Tilt-down peaks are more pronounced, probably because of the non-symmetric and slower intervention of parasympathetic activity with respect to penalization of the sympathetic activity regulating baroreflex and cardio-pulmonary reflex control. For instance, consider the case of brachial mean arterial pressure $\bar{p}$: it shows almost monotonic increase under tilt-up to 70° at 1.4°/s, with barely visible oscillation after the first ramp ascent; differently, $\bar{p}$ response to tilt-down from 70° is characterized by a first evident peak followed by a consequent decrease to the steady-state supine value.

**Limitations**. The main limitation of the presented model concerns its short-term focus, lacking longer time scale mechanisms such as chemical, metabolic, and hormonal regulations. Further adaptation regarding larger time scale compensatory mechanisms was not considered in this work. In addition, capillary filtration (transcapillary blood flow) is accounted for by some authors, although the long time scale nature of such mechanisms justifies our choice not to include them. Venous collapsibility—which is especially important for jugular veins when assuming the upright posture—is not implemented here. Despite a model of cerebral autoregulation being implemented in our work, no detailed modeling of the cerebral circulation is provided. Muscular activation is not taken into account, thus, only passive assumption of different tilted postures can be simulated.

## 5. Conclusion

In conclusion, multiscale modeling represents a powerful way of investigating cardiovascular global and specific response to the change of posture, providing feasible diagnostic solutions to inquire into single cause-effect relationships, excluding the intervention of concurrent mechanisms involved in the complex human CVS functioning and regulation.

The main findings of our study are summarized in Table 3. Our model showed that passive upright tilt causes an overall increase of mean arterial pressure, heart rate, and peripheral resistance, and a decrease of stroke volume, cardiac output, and central venous pressure. Pressure and flow rate waveform analysis along the arterial tree, together with mechano-energetic (stroke work) and oxygen consumption (time-tension index) parameters suggest that the CVS reaches a less stressed condition at passive upright posture than supine, with slight impairment of the energy supply-demand ratio. The tilt-up vs. down transient dynamic response is not symmetric and is non-linearly affected by the tilting rate, showing stronger under- and overshoots of the hemodynamic parameters as the duration of tilt is reduced.

**Table 3:** The main findings of our analysis on passive assumption of upright posture at different degree and rate of tilt.

**Focus**	**Results**	**Evidences**
Steady-state response	• MAP (brachial), HR, TPR increase; • SV, CO, CVP decrease; • wave phase shifting; • pulse pressure, SW (more), TTI (less) decrease; • passive upright posture is less energy-demanding than supine; • energy supply-demand imbalance; • central arterial hemodynamics more influenced than peripheral.	Table 2 Figures 3, 5–7
Transient response	•faster tilting enhances strong hemodynamic parameters under-/overshoots; • hemodynamic parameters monotonic behavior depending on rate of tilt; • not symmetric response between tilt up and down.	Figures 4, 8, 9

In view of future developments, by enriching the CVS response to posture changes, the present modeling approach can be exploited to investigate a number of applications: on Earth (such as autonomic dysfunction in the elderly, comparison with active standing, different exercise protocols, etc.) and during spaceflights (such as parabolic flight, short-term microgravity exposure, ground-based analogs and optimization of the current countermeasures), where clinical measurements—difficult and expensive to be performed—are overall few so far.

## Data Availability Statement

The raw data supporting the conclusions of this article will be made available by the authors upon request, without undue reservation.

## Ethics Statement

The studies involving human participants were reviewed and approved by Comitato Etico Interaziendale A.O.U. Città della Salute e della Scienza di Torino-A.O. Ordine Mauriziano-A.S.L. TO1-CEI/330. The patients/participants provided their written informed consent to participate in this study.

## Author Contributions

MF, LR, and SS conceived and designed the research, analyzed and interpreted the results, edited, and revised the manuscript. SM, MG, and MV performed the clinical experiments. MF performed the numerical simulations and contributed to the clinical campaign. MF drafted the manuscript and prepared the figures. All authors reviewed and approved the final version of the manuscript.

## Conflict of Interest

The authors declare that the research was conducted in the absence of any commercial or financial relationships that could be construed as a potential conflict of interest.

## Publisher's Note

All claims expressed in this article are solely those of the authors and do not necessarily represent those of their affiliated organizations, or those of the publisher, the editors and the reviewers. Any product that may be evaluated in this article, or claim that may be made by its manufacturer, is not guaranteed or endorsed by the publisher.
