# Supplementary material for: Cardiovascular Response to Posture Changes: Multiscale Modeling and in vivo Validation During Head-Up Tilt
Source: Front Physiol. 2022 Feb 17;13:826989. doi: 10.3389/fphys.2022.826989 (PMC8892183; doi:10.3389/fphys.2022.826989)
Supplement: Supplementary file 1 [file Data_Sheet_1.PDF]

Supplementary Material

SM 1 MATHEMATICAL MODEL

1D Arterial Tree. The overall model is illustrated in Figure S1. Data about the 1D arterial tree are reported in Table S1. Blood motion is described by the 1D axisymmetric form of the Navier-Stokes equations for mass and momentum balance:

$$\frac{\partial A}{\partial t} + \frac{\partial Q}{\partial x} = 0\,, \tag{S1}$$

$$\frac{\partial Q}{\partial t} + \frac{\partial}{\partial x}\left(\beta \frac{Q^2}{A}\right) + \frac{A}{\rho}\frac{\partial p}{\partial x} + N_4 \frac{Q}{A} + Ag \sin\gamma \sin\alpha = 0\,, \tag{S2}$$

where the dependent variables $A(x,t)$ and $Q(x,t)$ represent vessel lumen and blood flow rate, respectively, t is the time and x the vessel axial coordinate. Blood is modeled as a Newtonian fluid with density $\rho = 1050\, Kg/m^3$ and dynamic viscosity $\mu = 0.004\, Pa\, s$. A flat-parabolic velocity profile is assumed in order to derive analytic expressions for the Coriolis coefficient β and the viscous coefficient N_4.

Gravity effects are included via the source term $g \sin\gamma \sin\alpha$, where $g = 9.81\, m/s$ is the gravity acceleration, γ is the orientation of the vessel with respect to the frontal transverse body axis (γ values reported in Table S1, positive values indicates feetwards oriented vessels), and α is the vessel inclination with respect to the horizontal reference (tilt angle).

The constitutive equation for pressure $p(x,t)$ included to close the system (S1-S2) reads

$$p = B_1 + B_2 A + B_3 A^2 + B_4 A^3 - B_5 \frac{1}{\sqrt{A}}\frac{\partial Q}{\partial x}\,, \tag{S3}$$

where coefficients B_i ($i = 1 \ldots 5$) are function of vessels' geometry and mechanical properties, through the Pulse Wave Velocity (PWV).

Concerning boundary conditions, mass and total pressure conservation are imposed at inlet/outlet sections of arterial bifurcation points:

$$Q_{in} = Q_{out,1} + Q_{out,2}\,, \tag{S4}$$

$$p_{in} + \frac{1}{2}\rho\left(\frac{Q}{A}\right)^2_{in} = p_{out,1} + \frac{1}{2}\rho\left(\frac{Q}{A}\right)^2_{out,1} = p_{out,2} + \frac{1}{2}\rho\left(\frac{Q}{A}\right)^2_{out,2}\,, \tag{S5}$$

where subscripts in, $out,1$ and $out,2$ denote the parent vessel and the corresponding two daughter vessels (three for the coronary bifurcation) originating from a bifurcation, respectively. As external conditions

applied at the entrance of the aorta and at each terminal branch, a 0D model of the aortic valve is coupled with the proximal aorta, whereas a 0D distal arteriolar compartment is plugged to each terminal 1D distal artery, through a set of lumped characteristic impedances ($Z_c = \rho PWV/A_{out}$) defined for each terminal branch. Notice that values of Z_c pertaining to arms (#ids: 8, 10, 11, 22, 24, 25) and legs (#ids: 43, 45, 47, 48) regions have been reduced by 30% and augmented by 70%, respectively, with respect to previous model calibration, in order to match reported upright arterial pressure levels. Beside physical boundary conditions at the entrance of the aorta, at each terminal 1D artery and at arterial bifurcations, additional compatibility conditions were derived through the method of characteristics to deal with each boundary value problem (for further mathematical details on this aspect, refer to [13; 11]).

0D Systemic Microvasculature and Venous Return. Lumped parameter compartments representing systemic distal circulation, microcirculation and venous return are organized as shown in the bottom panel of Figure S1. The 0D arteriolar compartments connected to each 1D terminal artery are grouped into 5 major regions corresponding to separate body portions: head, arms, upper abdomen, lower abdomen and legs (referred to as H, A, UA, LA and L). One capillary (cap), one venular (ve), and one venous (v) compartments are defined for each region. Three additional districts are included to represent the superior (svc), inferior (ivc), and abdominal (avc) venae cavae.

Governing equations for the (i, j)-th 0D model read

$$\frac{dV_{i,j}}{dt} = Q_{i,j-1} - Q_{i,j}\,, \tag{S6}$$

$$\frac{dQ_{i,j}}{dt} = \begin{cases} \dfrac{p_{i,j} + \Delta p^h_{i,j} - R_{i,j}Q_{i,j} - p_{i,j+1}}{L_{i,j}}, & \text{if } j \in \{v, svc, ivc, avc\} \\ \dfrac{p_{i,j} - R_{i,j}Q_{i,j} - p_{i,j+1}}{L_{i,j}}, & \text{if } j \in \{art, cap, ven\} \end{cases}\,, \tag{S7}$$

where subscript i corresponds to the pertaining body region (H, A, UA, LA, L, notice that venae cavae compartments do not pertain to any of these body regions, *i.e.* no index i is assigned), whereas j denotes the compartment (art, gathering all arterioles connected to the considered body region, cap, ven, v, svc, and ivc, following avc). Intraluminal pressure of the (i, j)-th compartment is indicated with $p_{i,j}$, blood flow rate is denoted as $Q_{i,j}$, with $p_{i,j+1}$ and $Q_{i,j-1}$ being the blood pressure and flow rate belonging to the following and to the previous 0D compartments, respectively. $V_{i,j}$ is the compartmental total blood volume, obtained as the summation of the corresponding unstressed blood volume $V^{un}_{i,j}$ and the stressed blood volume. This latter depends on the compartmental transmural pressure $p^t_{i,j} = p_{i,j} - p^{ext}_{i,j}$ and on the associated compliance $C_{i,j}$ according to the following constitutive law

$$V_{i,j} = V^{un}_{i,j} + p^t_{i,j}C_{i,j} = V^{un}_{i,j} + \left(p_{i,j} - p^{ext}_{i,j}\right)C_{i,j}\,, \tag{S8}$$

where symbol $p^{ext}_{i,j}$ denotes either intrathoracic, intracranial or intramyocardial pressure (where no external pressure is specified, with respect to Figure S1, an external environmental relative pressure of 0 Pa is

assumed). The different constitutive law adopted to resemble non-linear effects of legs' veins pressure-volume relation reads

$$V_{L,v} = V_{L,v}^{un} + \frac{2\Delta V_{max}}{\pi} \arctan\left(\frac{\pi C_{L,v}}{2\Delta V_{max}} p_{L,v}^{t} \right), \tag{S9}$$

in which ΔV_{max} is the maximum distending volume of leg veins, assumed as high as 1200 ml [15; 14], $C_{L,v}$ is the leg venous compliance at zero transmural pressure (taken equal to venous compliance in supine position), and $p_{L,v}^{t} = p_{L,v}$ for leg venous transmural pressure. An additional relation can be derived for blood pressure $p(t)$ by combining equations (S8) and (S6):

$$\frac{dp_{i,j}}{dt} = \frac{Q_{i,j-1} - Q_{i,j}}{C_{i,j}}. \tag{S10}$$

All 0D parameter values are listed in Tables S2 and S3, ordered by body region and compartment.

Stevino's law is exploited to express hydrostatic pressure terms $\Delta p_{i,j}^{h}$:

$$\Delta p_{i,j}^{h} = \rho g \Delta h_{i,j} \sin\alpha, \tag{S11}$$

involving blood specific weight ρg, the hydrostatic height of the corresponding fluid column $\Delta h_{i,j}$ (only for the concerned compartments, *i.e.* $j \in \{v, svc, ivc, avc\}$), and the relative orientation with respect to the horizontal reference α (tilt angle). Due to the feetwards orientation of the body axis, compartments hydrostatic heights are taken as positive when moving accordingly to a head-feet direction (head veins, superior vena cava) whereas negative on the opposite direction (leg and arm veins, abdominal and inferior venae cavae). Blood column hydrostatic heights are associated with the anatomical length of the corresponding compartments projected along the body axis. They are determined as the summation/subtraction of half the characteristic lengths of two adjacent compartments, representing the vertical anatomical distance separating their compartmental midpoints. Assumed hydrostatic column heights are reported in Table S4.

0D Cardiopulmonary Circulation. Time-varying elastances $E_{ch}(t)$ are employed to model cardiac chambers mechanics. The equation linking cardiac transmural pressure $p_{ch}^{t} = p_{ch} - p^{ext}$ (where $p^{ext} = p^{it}$ is the intrathoracic pressure) and stressed volume $V_{ch} - V_{ch}^{un}$ (V_{ch}^{un} is the chamber's unstressed volume) reads

$$p_{ch} = E_{ch}\big(V_{ch} - V_{ch}^{un}\big) + p^{it}, \tag{S12}$$

whereas the elastance function $E_{ch}(t)$ is governed by the relation:

$$E_{ch} = E_{ch,A} e_{ch} + E_{ch,B}, \tag{S13}$$

where parameters $E_{ch,A}$ and $E_{ch,B}$ are chamber's elastance amplitude and baseline value, respectively. For the normalized shape-elastance function $e_{ch}(t)$ a different formulation is adopted for atria ($e_a(t)$):

$$e_a(t) = \begin{cases} \frac{1}{2}\left[1+\cos\left(\pi\frac{t+RR-t_{ar}}{T_{ar}}\right)\right], \; 0 \le t \le t_{ar}+T_{ar}-RR \\ 0\,, \qquad t_{ar}+T_{ar}-RR < t \le t_{ac} \\ \frac{1}{2}\left[1-\cos\left(\pi\frac{t-t_{ac}}{T_{ac}}\right)\right], \; t_{ac} < t \le t_{ac}+T_{ac} \\ \frac{1}{2}\left[1+\cos\left(\pi\frac{t-t_{ar}}{T_{ar}}\right)\right], \; t_{ac}+T_{ac} < t \le RR \end{cases}, \tag{S14}$$

and for ventricles ($e_v(t)$):

$$e_v(t) = \begin{cases} \frac{1}{2}\left[1-\cos\left(\pi\frac{t}{T_{vc}}\right)\right], \; 0 \le t \le T_{vc} \\ \frac{1}{2}\left[1+\cos\left(\pi\frac{t-T_{vc}}{T_{vr}}\right)\right], \; T_{vc} < t \le T_{vc}+T_{vr} \\ 0\,, \qquad T_{vc}+T_{vr} < t \le RR \end{cases}, \tag{S15}$$

where RR is the heart beat duration (*i.e.* $60/HR$, with HR the heart rate), parameters T_{ac}, T_{ar}, T_{vc} and T_{vr} correspond to atria and ventricles contraction and relaxation periods, respectively, and t_{ac} and t_{ar} denote atria contraction and relaxation starting time. All cardiac settings are summarized in Table S3.

By differentiating equation (S12), the chambers' pressure differential equation is obtained:

$$\frac{dp_{ch}}{dt} = \frac{dE_{ch}}{dt}\big(V_{ch} - V_{ch}^{un}\big) + E_{ch}\big(Q_{ch,in} - Q_{ch,out}\big) + \frac{dp^{it}}{dt}\,, \tag{S16}$$

where $Q_{ch,in}$ and $Q_{ch,out}$ are injected and ejected blood flow rate into and out of each cardiac chamber.

Cardiac valves are reproduced according to the model by Korakianitis *et al.* [22]:

$$L_{va}\frac{dQ_{va}}{dt} + R_{va}Q_{va} + B_{va}|Q_{va}|Q_{va} = \frac{\big(1-\cos(\theta_{va})\big)^4}{\big(1-\cos(\theta_{va,max})\big)^4}\big(p_{va,u} - p_{va,d}\big)\,, \tag{S17}$$

$$\begin{aligned} I_{va}\frac{d^2\theta_{va}}{dt^2} =& K_{p,va}\big(p_{va,u} - p_{va,d}\big) + K_{q,va}Q_{va}\cos(\theta_{va}) \\ & - K_{f,va}\frac{d\theta_{va}}{dt} - K_{v,va}Q_{va}\sin(2\theta_{va})\,. \end{aligned} \tag{S18}$$

Cardiac valves are described as non-ideal diodes accounting for several effects onto valve leaflets (affecting the valve opening angle θ_{va}), such as tissues friction ($K_{f,va}d\theta_{va}/dt$), pressure

$(K_{p,va}(p_{va,u} - p_{va,d}))$ and inertial $(K_{q,va}Q_{va}\cos(\theta_{va}))$ forces, and the influence of downstream vortexes $(K_{v,va}Q_{va}\sin(2\theta_{va}))$. B_{va} is Bernoulli's coefficient of the valve, R_{va} and L_{va} are valve resistance and inertance. Q_{va} is the flow across the valve, whereas $p_{va,u}$ and $p_{va,d}$ are upstream and downstream pressures acting onto valve leaflets (if $p_{va,u} - p_{va,d} \geq 0$ vortexes action is taken into account, otherwise it is discarded). Valve model parameters are summarized in Table S5 (k_p, k_q, k_f, k_v are coefficients K_{va} normalized by the valve's momentum of inertia I_{va}).

Arterial and venous pulmonary circulations are described by including a lumped resistance and a compliance each, as shown in Figure S1 (values reported in table S3).

Intrathoracic Pressure. A supine basal mean value of $p^{it}_{sup} = -2.5$ mmHg is assumed for intrathoracic pressure, whereas an upright mean value of $p^{it}_{up} = -6.5$ mmHg is reached after 90° tilt (reference [14]). The sinusoidal time oscillation about the mean value has been discarded for the sake of simplicity. Following Heldt [14], intrathoracic pressure mean value time variation during tilt is defined as

$$p^{it}(t) = \begin{cases} p^{it}_{sup}\,, & t < t_{tilt,0} \\ p^{it}_{sup} + \dfrac{p^{it}_{up} - p^{it}_{sup}}{2}\left(1 - \cos\left(\pi\dfrac{t - t_{tilt,0}}{\Delta t_{tilt}}\right)\right), & t_{tilt,0} \leq t \leq t_{tilt,0} + \Delta t_{tilt} \\ p^{it}_{up}\,, & t > t_{tilt,0} + \Delta t_{tilt} \end{cases}, \tag{S19}$$

where $t_{tilt,0}$ and Δt_{tilt} denote starting time and duration of head-up tilt procedure. Since, according to [14], intrathoracic pressure variation coincides with that assumed for the tilt angle α, the intrathoracic pressure dependency upon the tilt angle α is linear:

$$p^{it}(\alpha) = p^{it}_{sup} + \left(p^{it}_{up} - p^{it}_{sup}\right)\frac{\alpha}{90^\circ}\,. \tag{S20}$$

Multiscale Coronary Circulation. To define the multiscale representation of the coronary circulation, the same 1D description is employed for large coronary arteries (vessel numbered #49-62 in Figure S1 and Table S1), whereas the model introduced by Mynard *et al.* [32; 33] is adopted for the downstream coronary microvasculature (see left panel of Figure S1). Each 0D coronary microcirculatory district is subdivided into three branches referred to the three myocardial layers (subepicardium, midwall and subendocardium, denoted by index $jj = 1, 2, 3$ in Figure S1), in turn subdivided into an arterial, an intermediate and a venous compartment (index $ii = 1, 2, 3$). For the k-th 0D coronary microvascular district, $R_{ii,jj,k}$ are non-linear resistances (following Poiseuille's law), while $C_{ii,jj,k}$ and $V_{ii,jj,k}(t = 0)$ are compliances and initial blood volume. Murray's law is exploited to derive such parameters, as detailed in [40]. $Z_{a,k}$ and $Z_{v,k}$ are arterial and venous characteristic impedances, determined as $\rho PWV/A_{out}$, with a venous outlet radius 40% larger than the arterial one. Intramyocardial pressure is denoted as $p^{im}(t)$, accounting for the cavity-induced extracellular pressure (CEP), and for the shortening-induced intracellular pressure (SIP). All model settings and mathematical details are reported in [40]. Gravity effects are included within the 1D description of the coronary circulation only, while no hydrostatic pressure sources are enclosed in the terminal 0D circuital models, due to their limited anatomical extension.

Venous Valves. Venous valves (see [34; 21]) are represented through the definition of non-linear resistances $R_{A,v}$, $R_{L,v}$ and inertances $L_{A,v}$, $L_{L,v}$. Pressure balance across the arm/leg venous compartment (subscript $A/L, v$) is

$$\Delta p_{A/L,v} = R_{A/L,v} Q_{A/L,v} + B_{A/L,v} |Q_{A/L,v}| Q_{A/L,v} + L_{A/L,v} \frac{dQ_{A/L,v}}{dt} \,, \tag{S21}$$

where $\Delta p_{A/L,v} = p_{A/L,v} + \Delta p^h_{A/L,v} - p_{A/L,vc}$, being $p_{A/L,v} + \Delta p^h_{A/L,v}$ and $p_{A/L,vc}$ are pressures immediately upstream and downstream the venous valve. $B_{A/L,v} = \rho/(2A_{eff})_{A/L,v}$ is the Bernoulli's coefficient, while $L_{A/L,v} = \rho(l_{eff}/A_{eff})_{A/L,v}$ and $R_{A/L,v} = 8\pi\mu/(A_{eff})^2_{A/L,v}$, with l_{eff} assumed equal to the compartment radius, and the effective area A_{eff} defined as

$$A_{eff,A/L,v} = \big(A_{eff,max} - A_{eff,min}\big)_{A/L,v} \xi_{A/L,v}(t) + A_{eff,min,A/L,v} \,, \tag{S22}$$

through a function of the valve state $\xi_{A/L,v}$, expressed as

$$\xi_{A/L,v}(t) = \begin{cases} (1 - \xi_{A/L,v}) k_{vo} \Delta p_{A/L,v} \,, & \text{if} \quad \Delta p_{A/L,v} \geq 0 \\ \xi_{A/L,v} k_{vc} \Delta p_{A/L,v} \,, & \text{if} \quad \Delta p_{A/L,v} < 0 \end{cases} \,. \tag{S23}$$

In equations (S22-S23), $A_{eff,min,A/L,v} = 0$, $A_{eff,max,A/L,v} = A_{A/L,v}$, the latter expressed as $V_{A/L,v}/L_{A/L}$, involving compartmental total blood volume and characteristic length of the arms/legs venous compartments. From $A_{A/L,v}$ also compartment radius can be determined. Parameters $k_{vo} = k_{vc} = 40 mmHg^{-1}s^{-1}$ denote valve's opening and closing rate, respectively. Taking as reference the initial state of the system - for which $R_{A/L,v}(t=0) = R^0_{A/L,v}$ and $L_{A/L,v}(t=0) = L^0_{A/L,v}$, and with venous valves completely open ($A_{eff,A/L,v}(t=0) = A_{eff,max,a/l,v}$) - the following expressions can be derived for non linear resistance $R_{A/L,v}$ and inertance $L_{A/L,v}$:

$$R_{A/L,v} = R^0_{A,L,v} \frac{A^2_{eff,max,A/L,v}}{A^2_{eff,A/L,v}} \,, \qquad L_{A/L,v} = L^0_{A/L,v} \frac{A_{eff,max,A/L,v}}{A_{eff,A/L,v}} \,. \tag{S24}$$

Autonomic Control. Current aortic-carotid sinus pressure $\bar{p}_{acs}$ is determined by averaging pressure signals related to the three baroreflex sensors location (aortic arch $p_{AA}(t)$, right and left carotid sinus $p_{cs,R}(t)$ and $p_{cs,L}(t)$) over the RR cardiac cycle:

$$\bar{p}_{acs} = \frac{1}{3RR} \int_{RR} \big(p_{AA}(t) + p_{cs,R}(t) + p_{cs,L}(t)\big)\, dt \,. \tag{S25}$$

Then, sympathetic (n_s) and parasympathetic (n_p) activity are calculated as

$$n_s(\bar{p}_{acs}) = \frac{1}{1 + \left(\dfrac{\bar{p}_{acs}}{\bar{p}_{acs,tg}}\right)^{\nu}} \quad , \quad n_p(\bar{p}_{acs}) = \frac{1}{1 + \left(\dfrac{\bar{p}_{acs}}{\bar{p}_{acs,tg}}\right)^{-\nu}} \,, \tag{S26}$$

where, for arterial baroreflex, $\bar{p}_{acs,tg} = 84.34$ mmHg, and ν is the steepness of response, assumed equal to 7. Being $y_m(t)$ the generic efferent organ ($R_{i,art}$, $R_{i,cap}$, $V_{i,v}^{un}$, $V_{i,ve}^{un}$, $C_{i,v}$, $C_{i,ve}$, $E_{ch,max} = E_{ch,A} + E_{ch,B}$, HR), controlled by arterial baroreflex, its time evolution is governed by the following differential equation

$$\frac{dy_m}{dt} = \frac{1}{\tau_m}\Big(- y_m + \alpha_m n_s(\bar{p}_{acs}) - \beta_m n_p(\bar{p}_{acs}) + \gamma_m \Big) \, , \tag{S27}$$

and parameters α_m, β_m, γ_m and τ_m are reported in Table S6.

Low-pressure receptors are located into the right atrium, so that and estimation of the central venous pressure can be sensed. Target cardiopulmonary pressure $\bar{p}_{cp}$ is therefore

$$\bar{p}_{cp} = \frac{1}{RR}\int_{RR} p_{ra}(t)\, dt \, , \tag{S28}$$

with $n_{s,cp}(\bar{p}_{cp})$ and $n_{p,cp}(\bar{p}_{cp})$ defined as

$$n_{s,cp}(\bar{p}_{cp}) = \frac{1}{1 + \left(\dfrac{\bar{p}_{cp}}{\bar{p}_{cp,tg}}\right)^{\nu}} \quad , \quad n_{p,cp}(\bar{p}_{cp}) = \frac{1}{1 + \left(\dfrac{\bar{p}_{cp}}{\bar{p}_{cp,tg}}\right)^{-\nu}} \, , \tag{S29}$$

where reference pressure $\bar{p}_{cp,tg} = 7.21$ mmHg, and $\nu = 7$. Efferent organs control equation (S27) applies also for cardiopulmonary reflex. Parameters $\alpha_{cp,m}$, $\gamma_{cp,m}$ and $\tau_{cp,m}$ referred to variable $y_{cp,m}$ are enclosed in Table S6.

Cerebrovascular System. Typically, in supine position, intracranial pressure is as large as about 10 mmHg. As proposed in [17], starting from Davson's equation for intracranial pressure:

$$p^{ic} = R_{out} I_{form} + p_{dural} \, , \tag{S30}$$

where R_{out} and I_{form} are cerebrospinal fluid resistance and rate of formation, respectively, while p_{dural} identifies dural veins pressure, taken equal to central venous pressure (right atrium pressure) in supine position. For the tilted posture:

$$p^{ic}_{tilt} = R_{out} I_{form} + CVP_{tilt} - \Delta p^{h}_{H-ra} \, , \tag{S31}$$

where dural veins pressure has been approximated as central venous pressure $CVP = p_{ra}$ (right atrium pressure) at tilted posture, minus the head-right atrium gravity gradient according to Stevino's law ($\Delta p^{h}_{H-ra} = \rho g \Delta h_{H-ra} \sin\alpha$, with $\Delta h_{H-ra} = \Delta h_H + \Delta h_{svc}$, refer to Figure S2 and Table S4). By taking the difference between Davson's equation for tilted (S31) and supine (S30) position, the implemented relation governing p^{ic} with body position is obtained:

$$p^{ic}_{tilt} = p^{ic}_{sup} + CVP_{tilt} - CVP_{sup} - \Delta p^{h}_{H-ra} \, . \tag{S32}$$

To account for jugular vein collapsibility, only the gravity gradient associated with the fluid column extending from the head to the jugular point of collapse at zero pressure Δh_{H-ijv} is considered (here

represented by Δh_H, jugular vein assumed as corresponding to the superior vena cava compartment). The new relation implemented for tilt angles $\alpha \geq \alpha_{collapse}$ reads

$$p^{ic}_{tilt} = p^{ic}_{sup} - CVP_{sup} - \Delta p^{h}_{H-ijv}\,, \tag{S33}$$

where $\alpha_{collapse}$ is the angle for which $p_{svc} \geq 0$. Intracranial pressure has been introduced only for cerebral veins, as illustrated in Figure S1.

Cerebral Autoregulation. The model [52] consists of defining non-linear arteriolar compliances and resistances for cerebral vessels (v_{cer} numbered 6, 12, 13, 16, 17 and 20 in Figure S1 and Table S1) so that pressure equation S10 modifies as

$$\frac{dp_{H,art}}{dt} = \frac{Q_{H,a1Dout} - Q_{H,art}}{C_{H,art}} - \frac{p_{H,art}}{C_{H,art}}\frac{dC_{H,art}}{dt}\,, \tag{S34}$$

where $Q_{H,a1Dout}$ is the outlet blood flow coming from adjacent 1D cerebral arteries, and $dC_{H,art}/dt$ is given by

$$\frac{dC_{H,art}}{dt} = \frac{1}{\tau_{cer}}\Big(- C_{H,art} + \sigma(Gx_{cer})\Big)\,, \tag{S35}$$

where $x_{cer} = \big(\bar{Q} - \bar{Q}_{ref}\big)/\bar{Q}_{ref}$ is the feedback control parameter ($\bar{Q}_{ref} = 11.69$ ml/s) and $\bar{Q}$ is the mean current CBF, computed as

$$\bar{Q} = \frac{1}{RR}\int_{RR}\sum^{k\in v_{cer}} (Q_{H,a1Dout})_k\,dt\,. \tag{S36}$$

In equation (S35), parameter $G = 1.0$ ml/mmHg is the autoregulation maximum gain, $\tau_{cer} = 25s$ the autoreregulation time delay, and $\sigma(.)$ the sigmoidal activation function, defined as

$$\sigma(Gx_{cer}) = \frac{(C^0_{H,art} + \Delta C_{H,art}/2) + (C^0_{H,art} - \Delta C_{H,art}/2)e^{\frac{Gx_{cer}}{k_{cer}}}}{1 + e^{\frac{Gx_{cer}}{k_{cer}}}}\,, \tag{S37}$$

where $k_{cer} = \Delta C_{H,art}/4$ (defined in order to have $(d\sigma/dx)_{x=0} = -G$), whereas $\Delta C_{H,art}$ is determined as compliance variation between basal value $C^0_{H,art}$ and bounding values of maximum $C^{max}_{H,art} = 6C^0_{H,art}$ and minimum $C^{min}_{H,art} = 0.5C^0_{H,art}$ capacity. Then, depending on the feedback parameter sign:

$$\Delta C_{H,art} = \begin{cases} C^{max}_{H,art} - C^0_{H,art} = \Delta C_{H,art,1}\,, \text{ if } x_{cer} < 0 \\ C^0_{H,art} - C^{min}_{H,art} = \Delta C_{H,art,2}\,, \text{ if } x_{cer} \geq 0 \end{cases}\,. \tag{S38}$$

Non-linear arteriolar resistances $R_{H,art}$ are determined exploiting Poiseuille's law, taking as reference state arteriolar resistances and total blood volume at $t = 0$: $R^0_{H,art}$ and $V^0_{H,art}$, thus

$$R_{H,art} = R^0_{H,art} \left(\frac{V^0_{H,art}}{V_{H,art}} \right)^2 . \tag{S39}$$

Numerical Simulation. 1D governing equations were discretized and solved numerically according to a Discontinuous Galerkin Finite Elements approach, and integrated in time employing a 2-step Runge-Kutta explicit scheme [11] with constant time step. Ordinary differential equations governing 0D compartments were solved assuming blood pressure, $p(t)$, and flow rate, $Q(t)$, as dependent variables, computing blood volume, $V(t)$, by means of constitutive equations. Time integration was achieved via the same 2-step Runge-Kutta explicit scheme.

Time coupling between 0D and 1D models was obtained according to the following algorithm (further details in [11]):

1. compute 1D variables at the first half-step;
2. compute 0D variables at the first half-step;
3. compute boundary conditions (1D-0D interfaces and arterial bifurcations) at the first half-step;
4. compute 0D variables for the complete time step;
5. compute boundary conditions (1D-0D interfaces and arterial bifurcations) for the complete time step;
6. compute 1D variables for the complete time step.

The sequence of operations is repeated at each time step.

SM 2 HEAD-UP TILT TEST

Tilt test simulations are based on Heldt's [14] approach: tilt angle $\alpha(t)$, starting from the supine position $\alpha^{sup} = 0$ at $t_{tilt,0}$, reaches the desired tilted posture α^{up} within a given rotation period Δt_{tilt}. The $\alpha(t)$ implemented model is reported below:

$$\alpha(t) = \begin{cases} \alpha^{sup}, & t < t_{tilt,0} \\ \alpha^{sup} + \dfrac{\alpha^{up} - \alpha^{sup}}{2} \left(1 - \cos\left(\pi \dfrac{t - t_{tilt,0}}{\Delta t_{tilt}} \right) \right), & t_{tilt,0} \leq t \leq t_{tilt,0} + \Delta t_{tilt} \\ \alpha^{up}, & t > t_{tilt,0} + \Delta t_{tilt} \end{cases} . \tag{S40}$$

All tilted positions are reached keeping a constant tilting rate $\omega_{tilt} = 4°/s$, by varying the rotation period Δt_{tilt} accordingly, apart from the systematic study on the role of the tilting rate onto the acute response to change of posture. For this latter analysis, also different tilting velocities are simulated, respectively at 35°/s, 8°/s, 4°/s, 2.5°/s and 1.4°/s. Tilting starting time is defined $t_{tilt,0} = 60$ s, after completion of initial supine adjusting transient.

SM 3 MODEL VALIDATION

Model outcomes in terms of main haemodynamics alteration following HUT are presented in Tables S7a and S7b. Here, when possible, *in-vivo* clinical measurements - conducted in collaboration with *Azienda Ospedaliero-Universitaria Città della Salute e della Scienza di Torino* - are included (heart rate and pressures by Sphygmomanometers, stroke volume, cardiac output and peripheral resistance by Finometer MIDI) as $\mu \pm \sigma$, where, for each variable, σ is the overall data collection standard deviation from the ensemble mean μ, that is

$$\begin{aligned}\sigma^2 &= \frac{1}{N_1 + \dots + N_M}\Big(\sum_{i=1}^{N_1}\left(x_{i,1}-\mu\right)^2 + \dots + \sum_{i=1}^{N_M}\left(x_{i,M}-\mu\right)^2\Big) \\ &\approx \frac{1}{M}\Big(\sigma_1^2 + \dots + \sigma_M^2 + \left(\mu_1-\mu\right)^2 + \dots + \left(\mu_M-\mu\right)^2\Big),\end{aligned} \tag{S41}$$

where M is the number of subjects, x_i is the time series of the considered haemodynamic variable (time signal $p(t)$ and beat-to-beat parameters HR, SV etc.), and N denotes subjects sample size. The same relation is also applied for the computation of the ensemble average pressure signals standard deviation bands, from the single intra-subject waveform sequences acquisition.

In Tables S7a, S7b, studies concerning active standing at 90° are marked in order to distinguish them from passive tilt. Moreover, seated subjects are also put in evidence so that they can be separated from actual 90° standing.

In some works, also information about the transient during tilting test is available. All such references are marked in Tables S7a and S7b.

REFERENCES

[1]Antle, D., Cormier, L., Findlay, M., Miller, L., and Côté, J. (2018). Lower limb blood flow and mean arterial pressure during standing and seated work: Implications for workplace posture recommendations. *Preventive medicine reports* 10, 117–122. doi:https://doi.org/10.1016/j.pmedr.2018.02.016

[2]Asmussen, E. (1943). The distribution of the blood between the lower extremities and the rest of body. *Acta Physiologica Scandinavica* 5, 31–38. doi:https://doi.org/10.1111/j.1748-1716.1943.tb02030.x

[3]Blanco, P. and Feijóo, R. (2013). A dimensionally-heterogeneous closed-loop model for the cardiovascular system and its applications. *Medical Engineering & Physics* 35, 652–667. doi:https://doi.org/10.1016/j.medengphy.2012.07.011

[4]Blomqvist, C. G. and Stone, H. L. (1991). Cardiovascular adjustments to gravitational stress. *NASA. Lyndon B. Johnson Space Center, Spacelab Life Sciences 1: Reprints of Background Life Sciences Publications*

[5]Broomé, M., Maksuti, E., Bjällmark, A., Frenckner, B., and Janerot-Sjöberg, B. (2013). Closed-loop real-time simulation model of hemodynamics and oxygen transport in the cardiovascular system. *Biomedical engineering online* 12, 1–20. doi:doi={https://doi.org/10.1186/1475-925X-12-69}

[6]Coonan, T. and Hope, C. (1983). Cardio-respiratory effects of change of body position. *Canadian Anaesthetists' Society Journal* 30, 424–437. doi:https://doi.org/10.1007/BF03007869

[7]Critchley, L., Conway, F., Anderson, P., Tomlinson, B., and Critchley, J. (1997). Non-invasive continuous arterial pressure, heart rate and stroke volume measurements during graded head-up tilt in normal man. *Clinical Autonomic Research* 7, 97–101. doi:https://doi.org/10.1007/BF02267754

[8]Danielsen, M. and Ottesen, J. (2004). A baroreceptor model. In *Applied Mathematical Models in Human Physiology* (SIAM). 157–196

[9]Dell'Italia, L. and Walsh, R. (1988). Application of a time varying elastance model to right ventricular performance in man. *Cardiovascular research* 22, 864–874. doi:https://doi.org/10.1093/cvr/22.12.864

[10]Edgell, H., Robertson, A., and Hughson, R. (2012). Hemodynamics and brain blood flow during posture change in younger women and postmenopausal women compared with age-matched men. *Journal of applied physiology* 112, 1482–1493. doi:https://doi.org/10.1152/japplphysiol.01204.2011

[11]Gallo, C. (2021). *A multiscale modelling of the cardiovascular fluid dynamics for clinical and space applications*. Phd thesis, Politecnico di Torino

[12]Goswami, N., Batzel, J., Loeppky, J., and Hinghofer-Szalkay, H. (2011). Teaching fluid shifts during orthostasis using a classic paper by foux et al. *Advances in physiology education* 35, 330–335. doi:https://doi.org/10.1152/advan.00071.2011

[13]Guala, A., Camporeale, C., Tosello, F., Canuto, C., and Ridolfi, L. (2015). Modelling and subject-specific validation of the heart-arterial tree system. *Annals of biomedical engineering* 43, 222–237. doi:https://doi.org/10.1007/s10439-014-1163-9

[14]Heldt, T. (2004). *Computational models of cardiovascular response to orthostatic stress*. Ph.D. thesis, Massachusetts Institute of Technology. doi:http://hdl.handle.net/1721.1/28761

[15]Heldt, T., Shim, E., Kamm, R., and Mark, R. (2002). Computational modeling of cardiovascular response to orthostatic stress. *Journal of applied physiology* 92, 1239–1254. doi:https://doi.org/10.1152/japplphysiol.00241.2001

[16]Hinghofer-Szalkay, H. (2011). Gravity, the hydrostatic indifference concept and the cardiovascular system. *European journal of applied physiology* 111, 163–174. doi:https://doi.org/10.1007/s00421-010-1646-9

[17]Holmlund, P., Eklund, A., Koskinen, L., Johansson, E., Sundström, N., Malm, J., et al. (2018). Venous collapse regulates intracranial pressure in upright body positions. *American Journal of Physiology-Regulatory, Integrative and Comparative Physiology* 314, R377–R385. doi:https://doi.org/10.1152/ajpregu.00291.2017

[18]Ito, H., Kanno, I., Kato, C., Sasaki, T., Ishii, K., Ouchi, Y., et al. (2004). Database of normal human cerebral blood flow, cerebral blood volume, cerebral oxygen extraction fraction and cerebral metabolic rate of oxygen measured by positron emission tomography with 15 o-labelled carbon dioxide or water, carbon monoxide and oxygen: a multicentre study in japan. *European journal of nuclear medicine and molecular imaging* 31, 635–643. doi:https://doi.org/10.1007/s00259-003-1430-8

[19]Jose, A. and Collison, D. (1970). The normal range and determinants of the intrinsic heart rate in man. *Cardiovascular research* 4, 160–167. doi:https://doi.org/10.1093/cvr/4.2.160

[20]Karpeles, L. and Huff, R. (1955). Blood volume of representative portions of the musculoskeletal system in man. *Circulation research* 3, 483–489. doi:https://doi.org/10.1161/01.RES.3.5.483

[21]Keijsers, J., Leguy, C., Huberts, W., Narracott, A., Rittweger, J., and van de Vosse, F. (2016). Global sensitivity analysis of a model for venous valve dynamics. *Journal of biomechanics* 49, 2845–2853. doi:https://doi.org/10.1016/j.jbiomech.2016.06.029

[22]Korakianitis, T. and Shi, Y. (2006). Numerical simulation of cardiovascular dynamics with healthy and diseased heart valves. *Journal of biomechanics* 39, 1964–1982. doi:https://doi.org/10.1016/j.jbiomech.2005.06.016

[23]Leggett, R. and Williams, L. (1991). Suggested reference values for regional blood volumes in humans. *Health Physics* 60, 139–154. doi:https://doi.org/10.1088/1361-6560/aad313

[24]Liang, F., Takagi, S., Himeno, R., and Liu, H. (2009). Biomechanical characterization of ventricular–arterial coupling during aging: a multi-scale model study. *Journal of biomechanics* 42, 692–704. doi:https://doi.org/10.1016/j.jbiomech.2009.01.010

[25]Liang, F., Takagi, S., Himeno, R., and Liu, H. (2009). Multi-scale modeling of the human cardiovascular system with applications to aortic valvular and arterial stenoses. *Medical & biological engineering & computing* 47, 743–755. doi:https://doi.org/10.1007/s11517-009-0449-9

[26]Lim, E., Chan, G. S., Dokos, S., Ng, S., Latif, L. A., Vandenberghe, S., et al. (2013). A cardiovascular mathematical model of graded head-up tilt. *PloS one* 8, e77357. doi:https://doi.org/10.1371/journal.pone.0077357

[27]London, G., Weiss, Y., Pannier, B., Laurent, S., and Safar, M. (1987). Tilt test in essential hypertension. differential responses in heart rate and vascular resistance. *Hypertension* 10, 29–34. doi:https://doi.org/10.1161/01.HYP.10.1.29

[28]Ludbrook, J. (1962). Functional aspects of the veins of the leg. *American heart journal* 64, 706–713. doi:https://doi.org/10.1016/0002-8703(62)90257-0

[29]Malhotra, A., Cohen, D., Syms, C., and Townsend, R. (2002). Blood pressure changes in the leg on standing. *The Journal of Clinical Hypertension* 4, 350–354. doi:https://doi.org/10.1111/j.1524-6175.2002.00767.x

[30]Møller, S., Nørgaard, A., Henriksen, J., Frandsen, E., and Bendtsen, F. (2004). Effects of tilting on central hemodynamics and homeostatic mechanisms in cirrhosis. *Hepatology* 40, 811–819. doi:https://doi.org/10.1002/hep.1840400410

[31]Montero, D. and Rauber, S. (2016). Brain perfusion and arterial blood flow velocity during prolonged body tilting. *Aerospace medicine and human performance* 87, 682–687. doi:https://doi.org/10.3357/AMHP.4546.2016

[32]Mynard, J., Penny, D., and Smolich, J. (2014). Scalability and in vivo validation of a multiscale numerical model of the left coronary circulation. *American Journal of Physiology-Heart and Circulatory Physiology* 306, H517–H528. doi:https://doi.org/10.1152/ajpheart.00603.2013

[33]Mynard, J. and Smolich, J. J. (2015). One-dimensional haemodynamic modeling and wave dynamics in the entire adult circulation. *Annals of biomedical engineering* 43, 1443–1460. doi:https://doi.org/10.1007/s10439-015-1313-8

[34]Mynard, M., J Davidson, Penny, D., and Smolich, J. (2012). A simple, versatile valve model for use in lumped parameter and one-dimensional cardiovascular models. *International Journal for Numerical Methods in Biomedical Engineering* 28, 626–641. doi:https://doi.org/10.1002/cnm.1466

[35]Onizuka, C., Niimi, Y., Sato, M., and Sugenoya, J. (2015). Arterial blood pressure response to head-up tilt test and orthostatic tolerance in nurses. *Environmental health and preventive medicine* 20, 262–270. doi:https://doi.org/10.1007/s12199-015-0455-5

[36]Partsch, B. and Partsch, H. (2005). Calf compression pressure required to achieve venous closure from supine to standing positions. *Journal of vascular surgery* 42, 734–738. doi:https://doi.org/10.1016/j.jvs.2005.06.030

[37]Posch, A., Luippold, A., Mitchell, K., Bradbury, K., Kenefick, R., Cheuvront, S., et al. (2017). Sympathetic neural and hemodynamic responses to head-up tilt during isoosmotic and hyperosmotic hypovolemia. *Journal of neurophysiology* 118, 2232–2237. doi:https://doi.org/10.1152/jn.00403.2017

[38]Reymond, P., Merenda, F., Perren, F., Rufenacht, D., and Stergiopulos, N. (2009). Validation of a one-dimensional model of the systemic arterial tree. *American Journal of Physiology-Heart and*

Circulatory Physiology 297, H208–H222. doi:https://doi.org/10.1152/ajpheart.00037.2009

[39]Sagawa, K., Suga, H., Shoukas, A., and Bakalar, K. (1977). End-systolic pressure/volume ratio: a new index of ventricular contractility. *The American journal of cardiology* 40, 748–753. doi:https://doi.org/10.1016/0002-9149(77)90192-8

[40]Saglietto, A., Fois, M., Ridolfi, L., De Ferrari, G. M., Anselmino, M., and Scarsoglio, S. (2022). A computational analysis of atrial fibrillation effects on coronary perfusion across the different myocardial layers. *Scientific Reports* (in press)

[41]Senzaki, H., Chen, C., and Kass, D. (1996). Single-beat estimation of end-systolic pressure-volume relation in humans: a new method with the potential for noninvasive application. *Circulation* 94, 2497–2506. doi:https://doi.org/10.1161/01.CIR.94.10.2497

[42]Silvani, A., Calandra-Buonaura, G., Johnson, B., van Helmond, N., Barletta, G., Cecere, A., et al. (2017). Physiological mechanisms mediating the coupling between heart period and arterial pressure in response to postural changes in humans. *Frontiers in physiology* 8, 163. doi:https://doi.org/10.3389/fphys.2017.00163

[43]Smith, J., Porth, C., and Erickson, M. (1994). Hemodynamic response to the upright posture. *The Journal of Clinical Pharmacology* 34, 375–386

[44]Sprangers, R., Wesseling, K., Imholz, A., Imholz, B., and Wieling, W. (1991). Initial blood pressure fall on stand up and exercise explained by changes in total peripheral resistance. *Journal of Applied Physiology* 70, 523–530. doi:https://doi.org/10.1152/jappl.1991.70.2.523

[45]Stewart, J. and Montgomery, L. (2004). Regional blood volume and peripheral blood flow in postural tachycardia syndrome. *American Journal of Physiology-Heart and Circulatory Physiology* 287, H1319–H1327. doi:https://doi.org/10.1152/ajpheart.00086.2004

[46]Sundblad, P., Spaak, J., and Kaijser, L. (2014). Time courses of central hemodynamics during rapid changes in posture. *Journal of Applied Physiology* 116, 1182–1188. doi:https://doi.org/10.1152/japplphysiol.00690.2013

[47]Tahvanainen, A., Tikkakoski, A., Koskela, J., Nordhausen, K., Viitala, J., Leskinen, M., et al. (2016). The type of the functional cardiovascular response to upright posture is associated with arterial stiffness: a cross-sectional study in 470 volunteers. *BMC cardiovascular disorders* 16, 1–12. doi:https://doi.org/10.1186/s12872-016-0281-8

[48]Tanaka, H., Sjöberg, B., and Thulesius, O. (1996). Cardiac output and blood pressure during active and passive standing. *Clinical Physiology* 16, 157–170. doi:https://doi.org/10.1111/j.1475-097X.1996.tb00565.x

[49]Tansey, E., Montgomery, L., Quinn, J., Roe, S., and Johnson, C. (2019). Understanding basic vein physiology and venous blood pressure through simple physical assessments. *Advances in physiology education* 43, 423–429. doi:https://doi.org/10.1152/advan.00182.2018

[50]Toska, K. and Walløe, L. (2002). Dynamic time course of hemodynamic responses after passive head-up tilt and tilt back to supine position. *Journal of Applied Physiology* 92, 1671–1676. doi:https://doi.org/10.1152/japplphysiol.00465.2000

[51]Truijen, J., Kim, Y., Krediet, C., Stok, W., Kölgen, R., Colier, W., et al. (2012). Orthostatic leg blood volume changes assessed by near-infrared spectroscopy. *Experimental physiology* 97, 353–361. doi:https://doi.org/10.1113/expphysiol.2011.061051

[52]Ursino, M. and Lodi, C. (1997). A simple mathematical model of the interaction between intracranial pressure and cerebral hemodynamics. *Journal of Applied Physiology* 82, 1256–1269. doi:https://doi.org/10.1152/jappl.1997.82.4.1256

[53]van Campen, C., M, L., Verheugt, F., and Visser, F. (2018). Cerebral blood flow changes during tilt table testing in healthy volunteers, as assessed by doppler imaging of the carotid and vertebral arteries. *Clinical neurophysiology practice* 3, 91–95. doi:https://doi.org/10.1016/j.cnp.2018.02.004

[54]Van Lieshout, J., Harms, M., Pott, F., Jenstrup, M., and Secher, N. (2005). Stroke volume of the heart and thoracic fluid content during head-up and head-down tilt in humans. *Acta anaesthesiologica scandinavica* 49, 1287–1292. doi:https://doi.org/10.1111/j.1399-6576.2005.00841.x

[55]Vijaylakshmi, P., Veliath, S., and Mohan, M. (2000). Effect of head-up tilt on cardiovascular responses in normal young volunteers. *Indian journal of physiology and pharmacology* 44, 467–472

[56]Vrachatis, D., Papaioannou, T., Konstantopoulou, A., Nasothimiou, E., Millasseau, S., Blacher, J., et al. (2014). Effect of supine versus sitting position on noninvasive assessment of aortic pressure waveform: a randomized cross-over study. *Journal of human hypertension* 28, 236–241. doi:https://doi.org/10.1038/jhh.2013.101

[57]Youde, J., Panerai, R., Gillies, C., and Potter, J. (2003). Reproducibility of circulatory changes to head-up tilt in healthy elderly subjects. *Age and ageing* 32, 375–381. doi:https://doi.org/10.1093/ageing/32.4.375

[58]Zaidi, A., Benitez, D., Gaydecki, P., Vohra, A., and Fitzpatrick, A. (2000). Haemodynamic effects of increasing angle of head up tilt. *Heart* 83, 181–184. doi:http://dx.doi.org/10.1136/heart.83.2.181

SM 4 SUPPLEMENTARY FIGURES AND TABLES

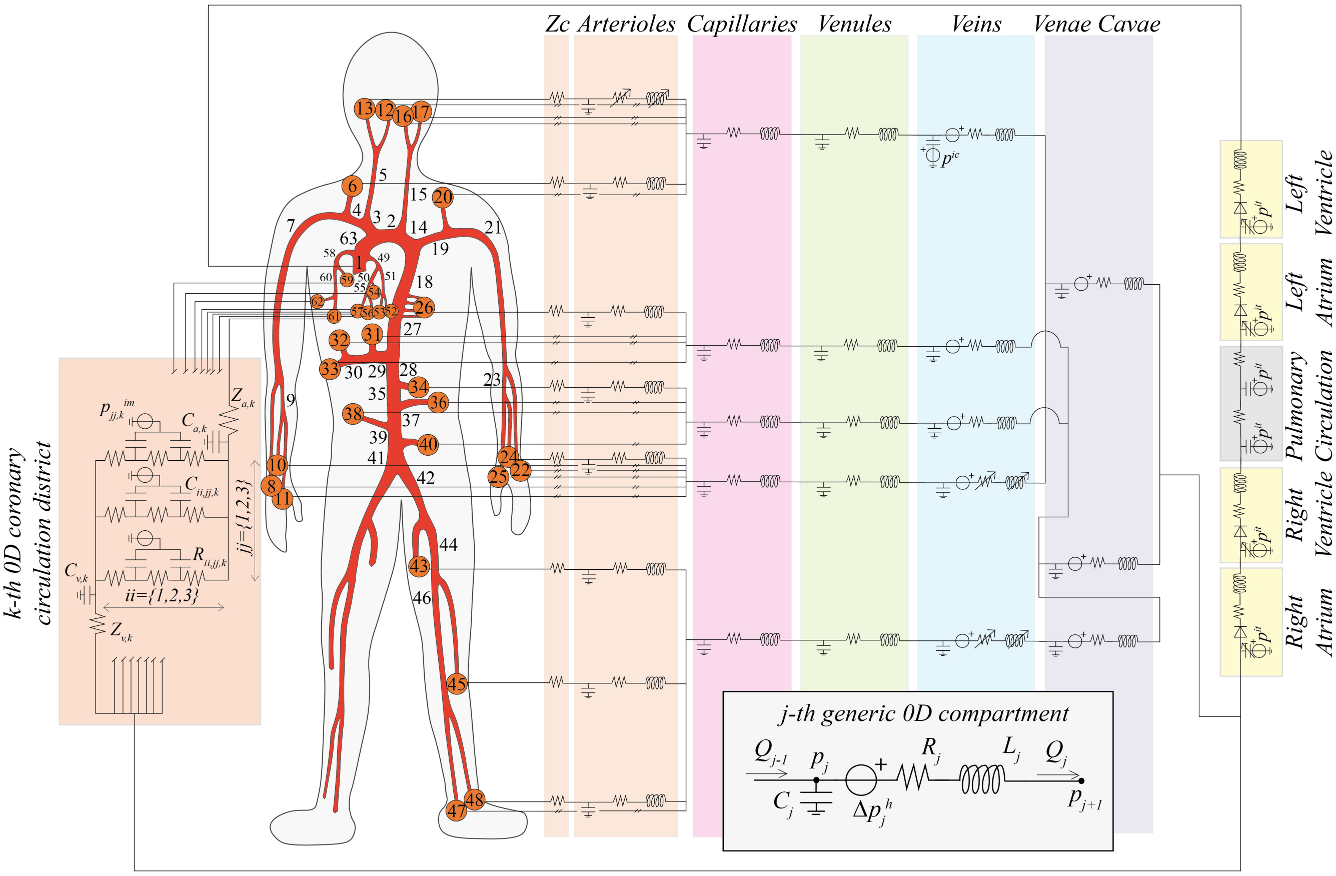

Figure S1. Complete illustration of the global multiscale cardiovascular model. The 1D arterial tree is depicted in red, black numbers refer to 1D main arterial vessels (see Table S1), numbers in orange circles denote terminal arteries connected to the downstream 0D compartments. Coloured boxes (orange, pink, green and blue, from left to right) subdivide the 0D peripheral circulation into arteriolar, capillary, venular, venous and venae cavae compartments. Z_c are arteriolar characteristic impedances, while R_j, C_j and L_j parameters, for the j-th 0D compartment, indicate vessels' lumped resistance, compliance and inertance, respectively. Q_j and p_j are compartmental intravascular blood flow rate and pressure (Q_{j-1} and p_{j+1} refer to the previous/subsequent compartment), Δp_j^h is the gravitational hydrostatic contribution. In the right coloured boxes, the cardio-pulmonary circulation is represented, where valves are depicted as diodes. The k-th generic 0D coronary microvascular district is reported in the left coloured box: $Z_{a,k}$, $Z_{v,k}$, $C_{a,k}$ and $C_{v,k}$ are arterial and venous impedances and compliances, respectively, $R_{ii,jj,k}$ and $C_{ii,jj,k}$ are intra-layer resitances and compliances. External pressures p^{it}, p^{ic} and p^{im} are intrathoracic, intracranial and intramyocardial pressure, respectively.

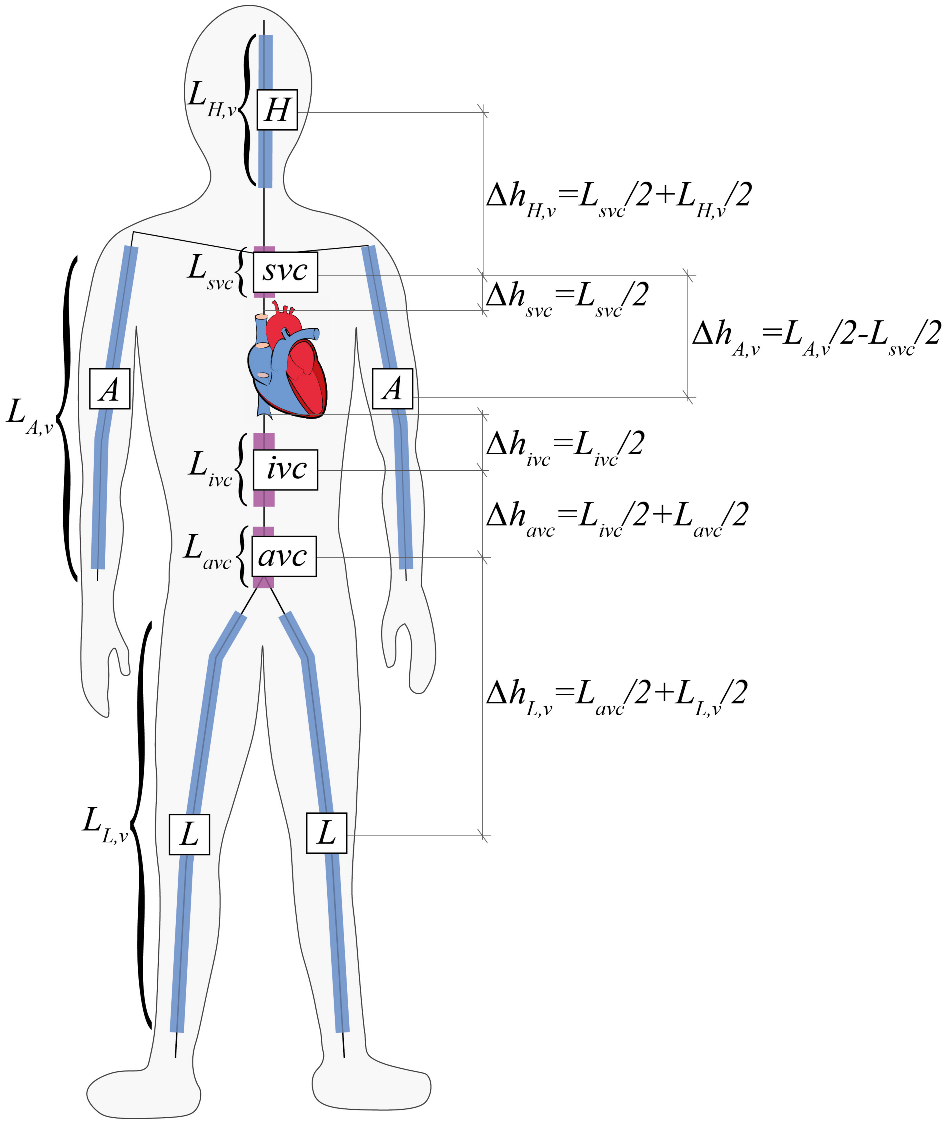

Figure S2. Venous and venae cavae vertical anatomical lengths organization. $L_{H,v}$, $L_{A,v}$, $L_{L,v}$, L_{svc}, L_{ivc} and L_{avc} are characteristic vertical anatomical lengths of the head, arms and legs veins, superior vena cava, inferior vena cava and abdominal vena cava compartments (reported in Table S4), $\Delta h_{H,v}$, $\Delta h_{A,v}$, $\Delta h_{L,v}$, Δh_{svc}, Δh_{ivc} and Δh_{avc} are vertical hydrostatic columns heights corresponding to the same compartments midpoint distances.

Table S1. Geometric properties of the 1D arterial tree (l: vessel length, D_{in} : vessel inlet diameter, D_{out}: vessel outlet diameter, h_{wall}: vessel wall thickness, γ: vessel anatomical orientation with respect to the frontal transverse body axis), data retrieved from Reymond et al. [38] and Blanco et al. [3]).

**#id**	**Artery**	l [mm]	D_{in} [mm]	D_{out} [mm]	h_{wall} [mm]	γ [°]
1	Ascending Aorta I	10	29.40	29.30	1.63	-90
2	Aortic Arch I	20	24.10	24	1.32	0
3	Brachiocephalic Artery	34	19.40	18	0.86	-45
4/19	R/L Subclavian Artery I	34	12.90/11	9/8.5	0.67	-30/-45
5/15	R/L Common Carotid Artery	94/139	15.10/12.40	7/6	0.63	-90
6/20	R/L Vertebral Artery	149/148	4.10/3.80	2.80	0.45	-90
7/21	R/L Subclavian II, Axillary & Brachial Arteries	422	8.90/8.40	4.70	0.66	+90
8/22	R/L Radial Artery	235	3.70/3.30	3.10/2.80	0.43	+90
9/23	R/L Ulnar Artery I	67	3.70/4	3.40/4	0.49	+90
10/24	R/L Interosseous Artery	79	2.10/1.80	1.80	0.28	+90
11/25	R/L Ulnar Artery II	171	3.20/4.10	2.80/3.70	0.49	+90
12/16	R/L Internal Carotid Artery	178	5.70/5	4.30/4.10	0.39	-90
13/17	R/L External Carotid Artery	41	5/4.50	4.50/4.10	0.42	-90
14	Aortic Arch II	39	22	20.80	1.27	0
18	Thoracic Aorta I	52	20	18.90	1.20	+90
26	Intercostal Arteries	80	12.60	9.50	1.20	0
27	Thoracic Aorta II	104	16.50	12.90	1.16	+90
28	Abdominal Aorta I	53	12.20	12.20	1.08	+90
29	Celiac Artery I	20	7.80	6.90	0.64	0
30	Celiac Artery II	25	5.20	4.90	0.64	0
31	Hepatic Artery	66	5.40	4.40	0.49	0
32	Gastric Artery	71	3.20	3	0.45	-90
33	Splenic Artery	63	4.20	3.90	0	
34	Superior Mesenteric Artery	59	7.90	7.10	0.69	+90
35	Abdominal Aorta II	20	11.50	11.30	0.8	+90
36/38	R/L Renal Artery	32	4.94	4.94/5.2	0.53	0
37	Abdominal Aorta III	20	11.20	11.20	0.8	+90
39	Abdominal Aorta IV	106	11	11	0.8	+90
40	Inferior Mesenteric Artery	50	4.70	3.20	0.43	+90
41	Abdominal Aorta V	20	10.80	10.40	0.8	+90
42	Common Iliac Artery	59	7.90	7	0.76	+45
43	Inner Iliac Artery	50	4	4	0.4	+45
44	External Iliac Artery	144	6.40	6.10	0.55	+90
45	Deep Femoral Artery	126	4	3.70	0.49	+90
46	Femoral Artery	443	5.20	3.80	0.5	+90
47	Anterior Tibial Artery	343	2.60	2.30	0.39	+90
48	Posterior Tibial Artery	321	3.10	2.80 6 0.45	+90	
49	Left Main Coronary Artery	12	4.50	4.50	0.4	0
50	Left Anterior Descending Coronary Artery I	18	3.74	3.74	0.4	+60
51	Circumflex Coronary Artery I	32	3.60	3.60	0.4	+60
52	Marginal Coronary Artery	73	2.52	2.52	0.3	+90
53	Circumflex Coronary Artery II	43	3	3	0.2	+90
54	Diagonal Coronary Artery	65	2.40	2.40	0.3	+90
55	Left Anterior Descending Coronary Artery II	22	3.29	3.29	0.3	+90
56	Septal Coronary Artery II	45	2.39	2.39	0.2	0
57	Left Anterior Descending Coronary Artery III	100	2.72	2.72	0.3	+90
58	Right Coronary Artery I	50	4.05	4.05	0.4	+60
59	Acute Marginal Coronary Artery I	34	2.52	2.52	0.3	0
60	Right Coronary Artery II	48	3.55	3.55	0.4	+90
61	Acute Marginal Coronary Artery II	23	2.11	2.11	0.2	+60
62	Right Coronary Artery III (Posterior Descending Artery)	89	3.21	3.21	0.3	+60
63	Ascending Aorta II	30	29.3	28.8	1.63	-90

Table S2. Lumped parameter arteriolar settings. R distal arteriolar resistance, C compliance, L inertance, V^{un} and V unstressed and total blood volume. Reference [11].

**Arteriolar #id**	R [mmHg s/ml]	C [mmHg/ml]	L [mmHg s/ml]	V^{un} [ml]	V [ml]
Vertebral 6/20	25.88	0.013	0.019	4.2	4.72
Radial 8/22	17.03	0.014	0.018	3.6	4.16
Interosseous 10/24	393.70	0.0009	0.070	3.6	3.63
Ulnar II 11/25	19.69	0.014	0.018	3.6	4.16
Internal Carotid 12/16	23.60	0.015	0.017	4.2	4.8
External Carotid 13/17	21.85	0.015	0.017	4.2	4.8
Intercostals 26	5.61	0.054	0.009	6.9	9.06
Hepatic 31	16.24	0.021	0.015	30.6	31.44
Gastric 32	8.91	0.033	0.012	9.3	10.62
Splenic 33	21.33	0.014	0.018	11.6	12.16
Superior Mesenteric 34	3.85	0.081	0.007	15.9	19.14
Renal 36/38	4.31	0.068	0.008	5.9	8.62
Inferior Mesenteric 40	30.74	0.011	0.02	11.9	12.34
Inner Iliac 43	23.48	0.014	0.018	3.5	4.06
Deep Femoral 45	13.37	0.023	0.014	14.4	15.32
Anterior Tibial 47	14.03	0.023	0.014	14.4	15.32
Posterior Tibial 48	30.44	0.010	0.021	14.4	14.80

Table S3. Lumped parameter values for systemic microcirculation and venous return. R distal arteriolar resistance, C compliance, L inertance, V^{un} and V unstressed and total blood volume. Body regions organized as head (H), arms (A), upper abdomen (UA), lower abdomen (LA) and legs (L). Venae cavae subdivided into superior (svc), inferior (ivc) and abdominal (avc) tracts. Pulmonary circulation splits into arterial (pa) and venous (pv) compartments. Parameters tuning based on [25; 24] with adjustments

**Compartment**	R [mmHg s/ml]	C [mmHg/ml]	L [mmHg s/ml]	V^{un} [ml]	V [ml]
		Capillary (cap)			
H	1.5166	0.0086	0.00049	7.49	7.75
A	1.7160	0.0058	0.00065	46.71	46.88
UA	0.9646	0.0121	0.00051	111.64	112.00
LA	0.5089	0.0226	0.00039	71.32	72.00
L	0.8682	0.0140	0.00037	56.86	57.28
		Venule (ve)			
H	0.4860	0.2560	0.00088	26.00	29.07
A	0.5494	0.1780	0.0012	173.66	175.80
UA	0.3088	0.4470	0.00093	414.64	420.00
LA	0.1622	0.6760	0.00096	261.89	270.00
L	0.2783	0.5500	0.00061	208.20	214.80
		Vein (v)			
H	0.2016	5.85	0.00098	60.08	60.08
A	0.2000	3.71	0.0013	333.64	363.32
UA	0.0594	49.50	0.0010	515.20	868.00
LA	0.0414	23.80	0.00078	367.60	558.00
L	0.1293	18.00	0.00067	299.92	443.92
		Vena Cava (vc)			
svc	0.0005	5	0.00005	30	60
ivc	0.0005	7.5	0.00005	55	100
avc	0.0005	7.5	0.00005	15	60
		Pulmonary			
pa	0.08	3.8	-	44.3	89.9
pv	0.005	20.5	-	232.8	396.8

Table S4. Characteristic vertical anatomical lengths of head, arms, lower/upper abdomen, leg veins and venae cavae, extracted from [15; 14; 26] or computed according to geometric proportions reported in Table S1.

Compartment	Vertical Length L [m]
H	0.15
A	0.55
UA	0
LA	0
L	1.00
svc	0.07
ivc	0.15
avc	0.07

Table S5. Parameter settings for the cardiac model, extracted from [11]. E_A, E_B are cardiac elastances amplitude and baseline values, V^{un}, V are unstressed and total chamber volumes, t_{ac}, T_{ac}, t_{ar}, T_{ar}, T_{vc}, T_{vr} denote atria and ventricles contraction/relaxation starting time and duration. R, L, B are valve's resistance, inertance and Bernoulli's coefficient, k_p, k_q, k_f, k_v indicate valve's geometric effects parameters (normalized by the valve's momentum of inertia).

**Chamber**	***ra***	***rv***	***la***	***lv***
E_A [ml/mmHg]	0.06	0.55	0.07	2.75
E_B [ml/mmHg]	0.14	0.08	0.11	0.10
V^{un} [ml]	6	12	6	7
V [ml]	91.9	116.3	109.9	109.4
t_{ac} [s]	$0.8RR$	-	$0.8RR$	-
T_{ac} [s]	$0.17RR$	-	$0.17RR$	-
t_{ar} [s]	$t_{ac} + T_{ac}$	-	$t_{ac} + T_{ac}$	-
T_{ar} [s]	T_{ac}	-	T_{ac}	-
T_{vc} [s]	-	$0.3\sqrt{RR}$	-	$0.3\sqrt{RR}$
T_{vr} [s]	-	$0.5T_{vc}$	-	$0.5T_{vc}$
**Valve**	***tv***	***pv***	***mv***	***av***
$R_{va} \cdot 10^{-6}$ [mmHg s/ml]	4.5	4.5	4.5	4.5
$L_{va} \cdot 10^{-6}$ [mmHg s/ml]	3.75	3.75	3.75	3.75
$B_{va} \cdot 10^{-6}$ [mmHg s^2/ml^2]	4.80	5.67	4.80	5.67
$k_{p,va}$ [rad/(mmHg s^2)]	5500	5500	5500	5500
$k_{q,va}$ [rad/(ml s)]	2	2	2	2
$k_{f,va}$ [rad/(ml s)]	50	50	50	50
$k_{v,va}$ [rad/(ml s)]	3.5	3.5	3.5	3.5

Table S6. Arterial baroreflex and cardiopulmonary reflex parameter settings. $E_{rv/lv,max}$ denotes maximum right/left ventricle elastance ($E_{rv/lv,A} + E_{rv/lv,B}$), subscript i for resistances, compliances and unstressed volumes indicates the corresponding body region (from head to legs). Each m-th organ-specific parameter is normalized by the initial value of the corresponding efferent organ variable (denoted by superscripts 0). Further details in [11; 8; 14].

**Arterial Baroreflex**				
y_m	α_m	β_m	γ_m	τ_m
HR/HR^0	0.75	0.75	1.00	5
$E_{rv/lv,max}/E^0_{rv/lv,max}$	0.40	-	0.80	5
$R_{i,art/cap}/R^0_{i,art/cap}$	5.00	-	-1.50	10
$C_{i,ve/v}/C^0_{i,ve/v}$	-0.60	-	1.30	30
$V^{un}_{i,ve/v}/V^{un,0}_{i,ve/v}$	-0.40	-	1.20	30
**Cardiopulmonary Reflex**				
$y_{cp,m}$	$\alpha_{cp,m}$	$\beta_{cp,m}$	$\gamma_{cp,m}$	$\tau_{cp,m}$
$R_{cp,i,art/cap}/R^0_{cp,i,art/cap}$	2.50	-	-0.25	15
$C_{cp,i,ve/v}/C^0_{cp,i,ve/v}$	-0.60	-	1.30	30
$V^{un}_{cp,i,ve/v}/V^{un,0}_{cp,i,ve/v}$	-0.40	-	1.20	30

Table S7a. comparisons between literature data (≊: no significant variation, - : no data available), our *in-vivo* findings (mean ± std.dev.) and model results for supine, HUT 30°, 70° and 90°. Asterisked superscripts *: transient information available, **: peripheral measure, not catheterized, ***: active standing, ****: subject seated, *****: typically surrogating the upright posture.

Variable	Supine			HUT 30°		
	Literature	Measured	Model	Literature	Measured	Model
Mean central arterial pressure [mmHg]	75÷110, [4], [16], [4], [27]	-	79	≊, [55]**,[50]*,**	-	83
Systolic central arterial pressure [mmHg]	100÷150, [4], [4], [27], [57]**	-	111	-1%÷-5%, [55]**	-	110 (-0.01%)
Diastolic central arterial pressure [mmHg]	60÷90, [4], [27], [57]**	-	63	+1%÷+7%, [55]**	-	70 (+11%)
Mean brachial arterial pressure [mmHg]	66÷105, [19], [7], [30]	85±10	82	-5%÷+23%, [58](45°)**, [7]*,**	86±8	89 (+9%)
Mean cerebral arterial pressure [mmHg]	75÷110, [4],[16]	-	84	-	-	82
Mean legs arterial pressure [mmHg]	75÷110, [4], [16]	93±10	85	110 (dia)÷175 (sys), [4],[45](35°)	141±16	135
Central venous pressure (right atrium) [mmHg]	0÷8, [49], [46],[4], [5]	-	7.2	-27%÷-36%, [46] (from transitory), [4]	-	4.3 (-40%)
Legs venous pressure [mmHg]	8÷10, [49],[28], [36]	-	9	27÷33, [45](35°)	-	34
Cerebral venous pressure [mmHg]	7÷10, [49]	-	9.4	-	-	0.9
Heart rate [bpm]	69÷77, [19], [58], [4], [4], [7], [27], [30], [14]	64±14	69	≊÷+13%, [37], [50]*, [58](45°), [55], [7]*	66±12	73 (+6%)
Stroke volume [ml]	59÷119, [4], [4], [58], [31], [7], [27], [30], [5]	121±20	75	-13%÷-25%, [50]*, [58](45°), [31], [7]*	100±16	61 (-19%)
Cardiac output [l/min]	3.9÷8.7, [4], [4], [58], [19], [31], [27], [30], [5]	7.7±3.2	5.2	-13%÷-17%, [50]*, [58](45°), [7], [31]	6.8±1.6	4.4 (-15%)
Cardio-pulmonary blood volume [ml]	800÷1170, [23], [27], [4]	-	880	-90÷-150, [45](35°)	-	767
Lower limbs blood volume [ml] (% of total blood volume)	680÷1197, [20], [2]	-	983	+4%÷+5%, [45](35°)	-	1231 (+4%)
Cerebral blood flow [ml/s]	9.5÷15, [4], [18]	-	10.9	≊, [31]*	-	10.7 (-2%)
Total peripheral resistance [mmHg s/ml]	0.79÷1.32, [4], [7], [30], [31], [27],[14]	0.55±0.10	0.83	+17%÷+28%, [50]*, [31], [7]*	0.65±0.13	1.08 (+30%)
Left max cardiac elastance [mmHg/ml]	1.8÷3.6, [41]	-	2.84	up to +25%, [39]	-	2.88 (+1.4%)
Right max cardiac elastance [mmHg/ml]	0.5÷2.1, [9]	-	0.63	up to +25%, [39]	-	0.64 (+1.5%)

Table S7b. (continue Table S7a) comparisons between literature data (≊: no significant variation, - : no data available), our *in-vivo* findings (mean ± std.dev.) and model results for supine, HUT 30°, 70° and 90°. Asterisked superscripts *: transient information available, **: peripheral measure, not catheterized, ***: active standing, ****: subject seated, *****: typically surrogating the upright posture.

Variable	HUT 70°,*****			HUT 90°		
	Literature	Measured	Model	Literature	Measured	Model
Mean central arterial pressure [mmHg]	≊, [54], [27](50°), [55](60°)**, [47](60°)**, [35]**	-	83	≊, [6]****, [43]*,***, [55](80°)**, [4]****, [56]***	-	83
Systolic central arterial pressure [mmHg]	105÷139, [43]*, [57]*,**, [55](60°)**, [47](60°)**, [27](50°)	-	105	97÷148, [6]****, [55](80°)**, [4]****, [56]***	-	106
Diastolic central arterial pressure [mmHg]	70÷88, [43]*, [57]*,**, [55](60°)**, [47](60°)**, [27](50°)	-	72	70÷109, [6]****, [55](80°)**, [4]****, [56]***	-	72
Mean brachial arterial pressure [mmHg]	-3%÷+33%, [58](60°,75°)**, [7](55°)*,**, [30](60°), [42](65°)**	89±7	90 (+10%)	97÷125, [44]*,***, [58]**, [56]***, [48]**,***, [10]**,***	-	91
Mean cerebral arterial pressure [mmHg]	-	-	76	65÷90, [16],[6]****, [10]***	-	76
Mean legs arterial pressure [mmHg]	150 (dia)÷200 (sys), [4](75°)	179±17	173	140÷230, [29],[1]	-	179
Central venous pressure (right atrium) [mmHg]	0.4÷6.2, [4](75°), [54],[27](50°),[30](60°), [46](80°)	-	1.4	0÷2 (-50%÷-100%), [43]****	-	0.7
Legs venous pressure [mmHg]	75÷100 (feet), [4](75°)	-	55	40÷100, [4],[16]	-	58
Cerebral venous pressure [mmHg]	-	-	-7.0	-10÷0, [4],[16]	-	-8.4
Heart rate [bpm]	+7%÷+34%, [51]*, [44]*, [54], [43]*, [30](60°), [7](55°)*, [42](65°), [27](50°)	79±12	86 (+25%)	+15%÷+45%, [4]****, [44]*,***, [6], [58], [10]***, [48]***, [43]***	-	85 (+23%)
Stroke volume [ml]	-20%÷+40%, [51]*, [44]*, [54], [43]*, [30](60°), [7](55°)*, [27](50°)	82±18	50 (-33%)	-25%÷-50%, [49], [58], [44]*,***, [4]****, [6]****, [10]***, [48]***, [43]*,***	-	51 (-32%)
Cardiac output [l/min]	-4%÷-21%, [51]*, [44], [30](60°), [54], [43]*, [27](50°), [47](60°)	6.8±1.4	4.2 (-19%)	-15%÷-40%, [49], [58], [44]*,***, [4]****, [6]****, [10]***, [48]***, [43]*,***	-	4.3 (-15%)
Cardio-pulmonary blood volume [ml]	-78÷-360, [27](50°)	-	650	-200÷-500 (-2%÷-9%), [6]****, [43]*,***	-	638
Lower limbs blood volume [ml] (% of total blood volume)	+6%÷+9%, [51]*, [2](60°)	-	1386 (+7%)	+300÷+800 (+5%÷+15%), [51], [4],[12]*,[1]	-	1406 (+7%)
Cerebral blood flow [ml/s]	-2.6%÷-9.4%, [53]	-	10.5 (-4%)	up to -10%÷-20%, [6]****,[43]	-	11.5 (-4%)
Total peripheral resistance [mmHg s/ml]	+3%÷+44%, [51], [44]*, [27](50°), [10], [30](60°),[43]*, [7](55°)*, [47](60°)	0.71±0.24	1.15 (+39%)	+20%÷+60%, [6]****, [10]***, [44]*,***, [48]***, [43]*,***	-	1.15 (+39%)
Left max cardiac elastance [mmHg/ml]	up to +25%, [39]	-	3.02 (+6%)	up to +25%, [39]	-	3.01 (+6%)
Right max cardiac elastance [mmHg/ml]	up to +25%, [39]	-	0.67 (+6%)	up to +25%, [39]	-	0.67 (+6%)
